# Igf signaling couples retina growth with body growth by modulating progenitor cell division

**DOI:** 10.1242/dev.199133

**Published:** 2021-04-15

**Authors:** Clara Becker, Katharina Lust, Joachim Wittbrodt

**Affiliations:** 1 Centre for Organismal Studies, Heidelberg University, Heidelberg 69120, Germany; 2 Heidelberg Biosciences International Graduate School, Heidelberg 69120, Germany

**Keywords:** Medaka, Organ size, Scaling, Self-organization

## Abstract

How the body and organs balance their relative growth is of key importance for coordinating size and function. This is of particular relevance in organisms, which continue to grow over their entire life span. We addressed this issue in the neuroretina of medaka fish (*Oryzias latipes*), a well-studied system with which to address vertebrate organ growth. We reveal that a central growth regulator, Igf1 receptor (Igf1r), is necessary and sufficient for proliferation control in the postembryonic retinal stem cell niche: the ciliary marginal zone (CMZ). Targeted activation of Igf1r signaling in the CMZ uncouples neuroretina growth from body size control, and we demonstrate that Igf1r operates on progenitor cells, stimulating their proliferation. Activation of Igf1r signaling increases retinal size while preserving its structural integrity, revealing a modular organization in which progenitor differentiation and neurogenesis are self-organized and highly regulated. Our findings position Igf signaling as a key module for controlling retinal size and composition, with important evolutionary implications.

## Abstract (precis)

**Highlighted Article:** Targeted activation of Igf1r signaling in the retinal stem cell niche increases retina size through expanding the progenitor but not stem cell population.

## INTRODUCTION

During embryonic development and postembryonic life of multicellular organisms, the proportions of overall body and organ size are actively controlled, being increased or decreased in a species-specific manner. Scaling of overall body and organ size is known to be regulated by systemic signals, which couple nutritional status to growth (Andersen et al., 2013). Conversely, differential growth of organs can be the result of altered sensitivity to systemic signaling (Tang et al., 2011) as well as of variant intrinsic signaling in the respective organ (Bosch et al., 2017; Twitty and Schwind, 1931).

Different eye dimensions of teleost species have been shown to be of functional relevance, as visual acuity is notably correlated with eye size (Caves et al., 2017). Vision and in particular retinal size and relative cell type composition are highly relevant for habitat-specific function and consequently for speciation. Deep-sea fish retinae show striking structural differences to the retinae of surface-dwelling fish, accommodating their specific need of increased light sensitivity (Caves et al., 2017; Darwish et al., 2015; de Busserolles et al., 2014; Wagner et al., 1998). How retinal growth is regulated in different fish species to achieve differential eye sizes and function is not understood.

Teleost fish such as medaka (*Oryzias latipes*) and zebrafish (*Danio rerio*) are particularly appealing model systems for studying organ size scaling. They display life-long postembryonic growth and each organ harbors distinct stem cell populations, which continuously self-renew, generating progenitor and, ultimately, differentiated cells (Aghaallaei et al., 2016; Centanin et al., 2014; Furlan et al., 2017). In the eye, growth of lens, retina and cornea is governed by adjacent stem cell niches (Mochizuki et al., 2014; Nowell and Radtke, 2017; Raymond Johns, 1977), and the precise regulation of continuous growth is of utmost importance to ensure the functional consistency of optical parameters such as photoreceptor density, matching of the focal point, photoreceptor to ganglion cell convergence and, therefore, vision.

Retinal growth in teleosts and amphibians is coordinated by the neuroretinal stem cell niche: the ciliary marginal zone (CMZ) (Hollyfield, 1971; Johns and Easter, 1977; Raymond Johns, 1977; Straznicky and Gaze, 1971). Two proliferating cell populations in the CMZ are responsible for the majority of postembryonic neurogenesis: multipotent, slowly dividing self-renewing stem cells located at the outermost periphery of the CMZ, and rapidly dividing progenitor cells located closer to the differentiated retina (Centanin et al., 2014; Raymond et al., 2006; Tsingos et al., 2019; Wan et al., 2016). Both stem and progenitor cells represent suitable cell type sources for altering retinal size, as they continuously proliferate and likely possess signal transduction machinery necessary for extrinsic signaling modulation. Which signals and which of the two CMZ cell types coordinate retinal growth with overall body size could not be addressed due, in part, to the lack of cell-type specific drivers.

A central integrator of organ size within a growing organism is hormonal signaling, translating extrinsic conditions such as nutrient availability into proportionate and coordinated growth (Andersen et al., 2013; Boulan et al., 2015). The insulin signaling pathway plays a central role in regulating embryonic development and growth, and its key function is underlined by a single nucleotide polymorphism in *insulin-like growth factor 1* (*igf1*) as causative alteration in small dog breeds (Sutter et al., 2007). Similarly, mutations in components of the Igf signaling pathway or their knockdown lead to severe dwarfism phenotypes from fish to humans (Baker et al., 1993; Klammt et al., 2008; Liu et al., 1993; Schlueter et al., 2007). Ultimately, final organ size is specified by systemic signals and their organ-specific interpretation (Shingleton and Frankino, 2018). The expression and localization of Igf pathway components during teleost embryonic and larval development has been assessed in a variety of species (Ayaso et al., 2002; Boucher and Hitchcock, 1998; Radaelli et al., 2003a,b; Zygar et al., 2005). Consistently, Igf ligands and receptors (Igfr) were found in the developing and adult retina. *igf1* is expressed in the postembryonic goldfish retina, and its receptor is expressed in the CMZ and inner plexiform layer (IPL) (Boucher and Hitchcock, 1998; Otteson et al., 2002). In zebrafish, Igf1r-mediated signaling is required for proper embryonic development, especially of anterior neural structures, and inhibition results in reduced body size, growth arrest and developmental retardation (Eivers et al., 2004; Schlueter et al., 2007). Despite the expression of its pathway components in the CMZ, the role of Igf1r signaling in the teleost CMZ as well as the consequences of its alteration on size and shape of the continuously growing neuroretina have not been addressed.

In this study, we address the coordination of retinal size with body growth and show that they are coupled by Igf1r signaling in the postembryonic retinal stem cell niche of medaka. We find that global Igf inhibition reduces proliferation in the CMZ. Uncoupling Igf signaling between body and retina by targeted constitutive activation of Igf1r specifically in stem and progenitor cells of the neuroretina increases proliferation, leads to an increase in eye size and uncouples retinal size from body size. Importantly, the resulting oversized retinae are structurally intact and correctly laminated with expanded thickness of the retinal layers, indicating that progenitor differentiation and neurogenesis are self-organized. We demonstrate that Igf1r activation decreases cell cycle length in the CMZ, expands the progenitor population and ultimately leads to increased neuronal cell numbers. By specifically dissecting the individual contribution of stem cells and progenitor cells to the oversized retinae, we uncover that retinal progenitor, but not stem, cells integrate the modulation by Igf1r signaling.

## RESULTS

### Igf1r signaling regulates proliferation in the medaka CMZ

**Fig. 1.**
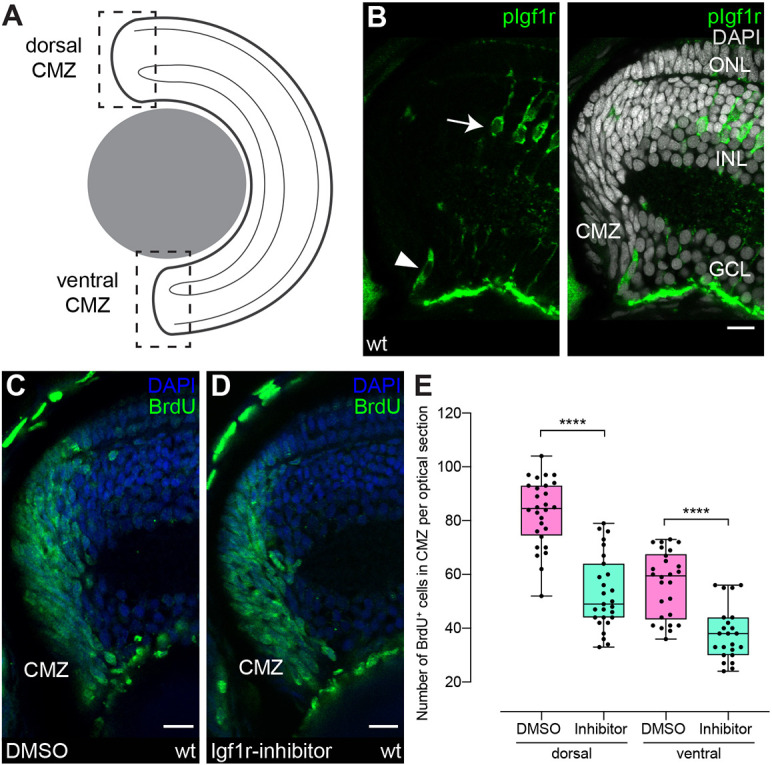
**Igf1r signaling regulates proliferation in the ciliary marginal zone.** (A) Schematic representation of a transverse retinal section. Dashed squares represent dorsal (d) and ventral (v) ciliary marginal zone (CMZ). All CMZ sections in this article depict the dorsal CMZ, with separate quantifications for dorsal and ventral CMZ, unless stated otherwise. (B) Cryosection of wild-type hatchling with anti-pIgf1r (green) staining shows Igf1r activity in single CMZ cells (arrowhead, *n*=35 cells in 15 sections from five fish) and in Müller glia (MG) (arrow) in the inner nuclear layer (INL). Scale bar: 10 μm. GCL, ganglion cell layer; ONL, outer nuclear layer. (C,D) Wild-type hatchlings were incubated for 24 h in BrdU and 10 μM Igf1r inhibitor NVP-AEW541 (D) or DMSO (C). Cryosections of DMSO- (C) and Igf1r inhibitor- (D) treated retinae with BrdU staining (green). Scale bars: 10 μm. (E) Quantification of BrdU-positive cell number in one optical section per central section shows a decrease in Igf1r-inhibitor-treated retinae [*n*=26 (dorsal)/23 (ventral) sections from 10 retinae in six fish] compared with DMSO [*n*=28 (dorsal)/27 (ventral) sections from 10 retinae in six fish] (data obtained from two independent experiments; *t*-test, ****P_d_<0.0001; Mann–Whitney test, ****P_v_<0.0001). Box plots show median, and 25th and 75th percentiles, with whiskers from minimum to maximum data points.

The retinal stem cell niche is located in a continuous ring in the CMZ at the periphery of the teleost eye (Fig. 1A). To address the involvement of the Igf signaling pathway in regulating retinal stem and progenitor cell proliferation in the medaka retina, we first assessed expression of receptors and ligands. We find *igf1ra*, *igf2* and *insrb* expressed in the CMZ, and in specific layers of the differentiated retina (Fig. S1).

The activity of receptor tyrosine kinases like Igf1r is mediated by ligand-induced conformational changes and subsequent trans-phosphorylation (Laviola et al., 2007). To assess whether Igf1r-mediated signaling is active in the CMZ, we detected the phosphorylated Igf1r (pIgf1r) by immunohistochemistry. Single cells in the progenitor domain of the CMZ (around 2%, estimation based on quantification of Pcna-positive cells in Fig. 4 and Fig. S5) as well as Müller glia (MG) cells in the inner nuclear layer (INL) in the differentiated part of the retina were positive for pIgf1r (Fig. 1B). The sparse distribution of pIgf1r-positive cells likely reflects a snapshot of the highly dynamic process of the activation and phosphorylation of receptor tyrosine kinases (Kiyatkin et al., 2020; Varkaris et al., 2013).

Igf1r expression and activity data in the CMZ are consistent with Igf signaling regulating proliferation in the CMZ to scale retinal size relative to the body. To determine the impact of Igf1r signaling on the proliferation of retinal stem and progenitor cells in the CMZ and on MG cells, we employed the widely used Igf1r inhibitor NVP-AEW541 (Chablais and Jazwinska, 2010; Choi et al., 2013; Huang et al., 2013) and BrdU to detect proliferatively active cells that entered or went through S phase during the incubation time. Although the NVP-AEW541 inhibitor displays a high specificity for Igf1r, an impact on Insr and other related kinases cannot be entirely ruled out (Attias-Geva et al., 2011; García-Echeverría et al., 2004). Fish at hatching stage [Iwamatsu stage 40 (Iwamatsu, 2004)] were incubated in 10 µM NVP-AEW541 or DMSO together with BrdU for 24 h, and analyzed subsequently by immunostaining against BrdU (Fig. 1C,D). Inhibitor incubation resulted in a 30% decrease of cells undergoing S phase in the CMZ (Fig. 1E), indicating that Igf/insulin signaling contributes to regulating proliferation in the CMZ. In the absence of MG cell proliferation in the differentiated retina, an impact of the inhibitor NVP-AEW541 was not detectable. Importantly, the 24 h incubation with the Igf1r inhibitor did not impact on relative eye size (Fig. S2A), nor did average eye diameter or body length differ between inhibitor treated and control groups. Moreover, due to the global nature of the inhibitor assay, a non-cell-autonomous effect of this treatment cannot be excluded. These results suggest that ligands and receptors of the Igf/insulin signaling cascade expressed in the CMZ contribute to CMZ proliferation.

### Constitutive activation of Igf1r in the CMZ results in increased neuroretinal size

Our results indicate that Igf1r signaling represents a likely target to coordinate and modulate retina-specific growth. To further test this hypothesis, we established and employed tools for uncoupling Igf1r signaling in the neuroretina from the rest of the body by the precise and constitutive activation of Igf1r signaling in the CMZ.

Activation of the Igf signaling pathway was achieved by a constitutively active *cd8a:igf1ra* chimeric receptor (*caigf1r*). We targeted its expression to stem and progenitor cells of the CMZ by employing the *rx2* promoter. The *cd8a:igf1ra* variant was generated by an in-frame fusion of the extracellular and transmembrane domain of medaka *cd8a* and the intracellular domain of medaka *igf1ra*, as previously described (Carboni et al., 2005). The *rx2* promoter drives expression in stem cells and early multipotent progenitors in the CMZ, as well as in MG and photoreceptor (PRC) cells in the differentiated retina (Reinhardt et al., 2015).

The potential of the caIgf1r fusion receptor to induce signaling transduction via the PI3K/Akt axis had been previously validated in several studies by assaying pAkt levels (Carboni et al., 2005; Gusscott et al., 2016; Pappano et al., 2009). Hence, we validated the functionality of the *rx2::caigf1r* transgenic line and addressed the activation of the signaling cascade downstream of Igf1r in *rx2*-positive cells by the phosphorylation status of its downstream effector Akt. In retinal sections of wild-type hatchlings, no pAkt staining was present in the peripheral domain in the CMZ (Fig. S2B). In contrast, *rx2::caigf1r* retinae showed prominent pAkt staining in the CMZ, overlapping with *caigf1r* expression in the *rx2* domain (Fig. S2C), and *rx2*-expressing MG cells and PRCs were also positive for pAkt. These results show that, in the retina of *rx2::caigf1r* transgenic fish, Akt is activated downstream of Igf1r in stem and progenitor cells in the CMZ, as well as in MG cells and PRCs in the differentiated retina.

**Fig. 2.**
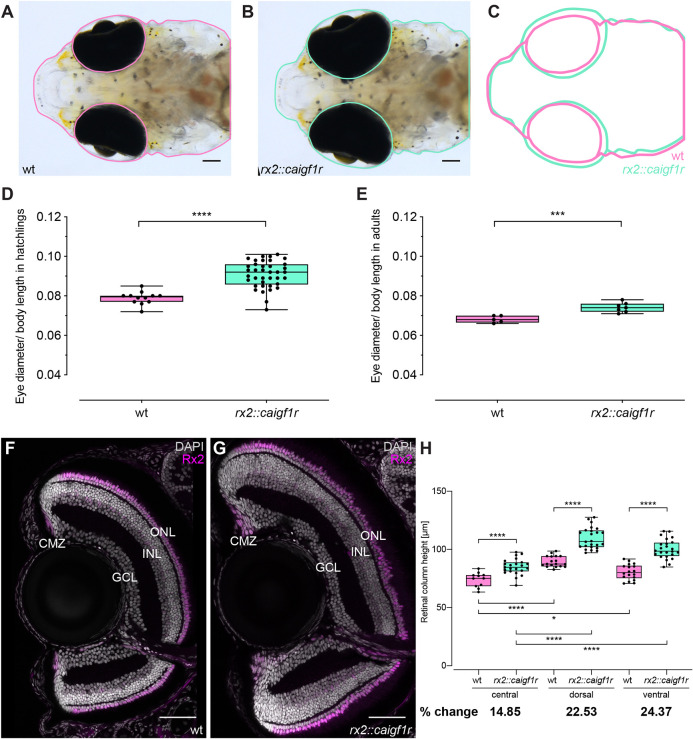
**Constitutive activation of Igf1r in retinal stem and progenitor cells results in increased neuroretina size.** (A-C) Eye size of *rx2::caigf1r* hatchlings (B, green in C) is increased compared with wild-type siblings (A, pink in C). Scale bars: 100 μm. (D,E) Quantification of relative eye size (eye diameter normalized to body length) of wild-type (D: *n*=12; E: *n*=11) and *rx2::caigf1r* (D: *n*=38; E: *n*=15) hatchlings (D), and 3-month-old adults (E) (*t*-test, ****P_hatchling_<0.0001, ***P_adult_=0.0009). (F,G) Cryosections of wild-type (F) and *rx2::caigf1r* (G) hatchling retinae with staining for Rx2 (magenta) display neuroretinal expansion. Wild-type section corresponds to fish 5 and *rx2::caigf1r* section to fish 16 in Table S1. Scale bars: 50 μm. (H) Neuroretinal thickness was measured perpendicular to the inner plexiform layer (IPL) in the fully laminated, CMZ-proximal region in wild-type and *rx2::caigf1r* retinae. Quantification of retinal column height in the central (wild type, *n*=11 sections from eight retinae in five fish; *rx2::caigf1r*, *n*=23 sections from ten retinae in eight fish), dorsal and ventral (wild type, *n*=18 sections from 12 retinae in six fish; *rx2::caigf1r*, *n*=24 sections from 14 retinae in eight fish) retina shows an increase in *rx2::caigf1r* compared with wild-type fish and in CMZ-derived compared with embryonic retina (*t*-test, ****P<0.0001, *P_wt c-v_=0.0130). Box plots show median, and 25th and 75th percentiles, with whiskers from minimum to maximum data points.

To address the potential of Igf1r signaling to modulate retinal growth, we next examined hatchlings for changes in retinal morphology related to *rx2::caigf1r* expression. Intriguingly, transgenic *rx2::caigf1r* hatchlings displayed an oversized retina compared with wild-type siblings (Fig. 2A-C), while other body parts remained unaffected. Relative eye size (anterior-posterior eye diameter normalized to body length) was increased in *rx2::caigf1r* hatchlings by 15% (Fig. 2D, Table S1), persisting throughout postembryonic growth until adulthood, where relative eye size was enlarged by 9% in *rx2::caigf1r* fish (Fig. 2E, Table S2). These data indicate that CMZ-targeted activation of Igf1r signaling uncouples retinal growth from overall body growth in medaka, disrupting the coordinated, Igf1r-mediated scaling of retina and body size.

Size increase of a tissue can arise due to different mechanisms of tissue expansion, such as increase in cell size or number, or stretching and increase in fluid or pressure (Ritchey et al., 2012; Stujenske et al., 2011; Veth et al., 2011). To understand how constitutive Igf1r signaling impacts on retina size, we examined cryosections of wild-type and *rx2::caigf1r* hatchling retinae. *Rx2::caigf1r* eyes exhibited a noticeable expansion of the neuroretina in all dimensions, in contrast to wild-type controls (Fig. 2F,G). Importantly, nuclear layer morphologies and arrangement indicated that the overall retinal architecture in *rx2::caigf1r* fish remained intact. The stereotypical structure of the neuroretina with the CMZ at the periphery, and three nuclear and two plexiform layers in the differentiated part was undisturbed by retinal expansion.

We further assessed retinal architecture using the expression of *rx2* as a landmark. All cell types expressing *rx2* in wild type are present in the oversized *rx2::caigf1r* retinae, both in the CMZ as well as centrally in MG cells and PRCs (Fig. 2F,G). To characterize the apparent neuroretinal expansion, we compared the neuroretinal thickness in the peripheral, fully laminated region in *rx2::caigf1r* and wild-type retinae, along a line perpendicular to the IPL. inner plexiform layer (IPL). Retinal column height was increased by 23-24% on average in the dorsal as well as the ventral retina (Fig. 2H). In addition, we measured height of the retinal column in central regions, reflecting the embryonic contribution of *rx2* expression in retinal progenitor cells to the oversized retinae. Column height of the central neuroretina in *rx2::caigf1r* hatchlings was increased by 15% compared with wild type (Fig. 2H). However, the increase was even more pronounced in peripheral, CMZ-derived regions, arguing for a greater impact of the post-developmental contribution via growth mediated by the CMZ.

To further substantiate our hypothesis that the increased size of the neuroretina is predominantly mediated by CMZ-derived cells, we assessed the relative contribution of the differentiated, central retinal region by proliferation analysis. A 2 h BrdU incubation of wild-type and *rx2::caigf1r* hatchlings yielded very low numbers of BrdU-positive cells per µm^2^ retinal area in both groups, with a slight elevation of proliferative MG cells and PRCs in oversized retinae reflecting a minor to negligible contribution to the apparent neuroretinal expansion (Fig. S3A-C). Moreover, we also excluded the possibility that, reduced apoptosis levels in the retina contributed to the size increase. TUNEL analysis revealed comparable numbers of apoptotic cells in the CMZ of wild-type and *rx2::caigf1r* hatchlings, and increased levels of TUNEL-positive cells in the central region of *rx2::caigf1r* retinae (Fig. S3D-F). This indicates that neither increased central proliferation nor decreased apoptosis is a major contributor to cell number expansion in *rx2::caigf1r* retinae. Taken together, these results show that the activation of Igf1r signaling in the CMZ increases retinal diameter and layer thickness, and the enlargement is achieved by a neuroretinal expansion through the increase in cell number rather than in cell size.

### Neuroretinal cell type composition is shifted toward INL neurons upon constitutive Igf1r activation

**Fig. 3.**
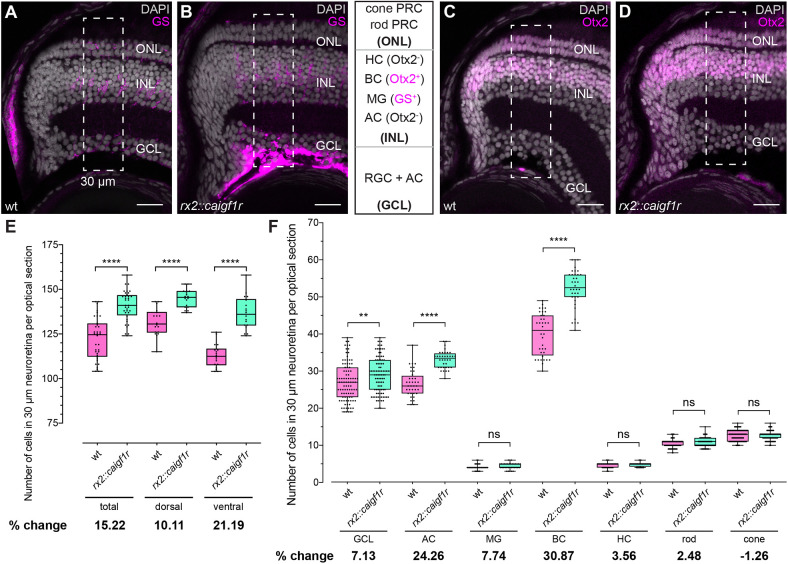
**Cell type composition of *rx2::caigf1r* retinae is shifted towards INL neurons.** (A-D) Cell type numbers were quantified in a 30 μm wide region (dashed rectangles) in the differentiated peripheral neuroretina in cryosections of wild-type (A,C) and *rx2::caigf1r* (B,D) hatchlings: cone and rod PRCs, and cells in the retinal ganglion layer (GCL) are identified by location; Müller glia (MG) are GS positive (A,B, magenta), bipolar cells (BCs) are Otx2 positive (C,D, magenta), and horizontal (HCs) and amacrine cells (ACs) are Otx2 negative (C,D). Scale bars: 20 μm. (E) Quantification of total cell number shows an increase in *rx2::caigf1r* [*n*=36 (total)/18 (dorsal/ventral) sections from six retinae in three fish] compared with wild-type [*n*=36 (total)/18 (dorsal/ventral) sections from six retinae in three fish] retinae (*t*-test, ****P<0.0001). The percentage change of mean cell number from wild type to *rx2::caigf1r* shows notable differences. (F) Quantification of the number of each cell type shows an increase in the number of GCL, ACs and BCs in *rx2::caigf1r* (*n*≥35 sections from six retinae in three fish) compared with wild-type (*n*≥36 sections from six retinae in three fish) retinae (*t*-test, ****P_AC_<0.0001; Mann–Whitney test, **P_GCL_=0.0076, ^ns^*P*_MG_=0.0724, ****P_BC_<0.0001, ^ns^*P*_HC_=0.3444, ^ns^*P*_rod_=0.1771 and ^ns^*P*_cone_=0.3114). Percentage change of mean cell number of wild type and *rx2::caigf1r* shows the largest changes in AC and BC number. ns, not significant. Box plots show median, and 25th and 75th percentiles, with whiskers from minimum to maximum data points.

The relative composition of retinal cell types reflects the functionality of the retina and differs considerably between teleost species. To elucidate whether in the oversized retinae cell type numbers were increased proportionally or whether cell type proportions were shifted, we analyzed the neuroretinal cell type composition in *rx2::caigf1r* and wild-type hatchlings in a 30 µm column spanning the entire neuroretinal column in the fully differentiated region proximal to the CMZ (dashed frame in Fig. 3A-D).

Cell type identity was determined by shape and position or by cell type-specific markers. PRCs are unambiguously characterized by their stereotypic localization in the outermost of the three nuclear layers. Retinal ganglion cells (RGCs) and displaced amacrine cells (ACs) located in the ganglion cell layer (GCL) were classified as one group. In the outer nuclear layer (ONL), cone PRCs were identified as outermost nuclear layer, and rods as the inner layer of the PRC nuclei. Other retinal cell types were distinguished by immunostaining with cell type-specific markers, such as GS, which labels MG cells, and Otx2, which labels bipolar cells (BCs) (Fig. 3A-D). Horizontal (HCs) and ACs in the INL were characterized as being Otx2 negative, together with their specific apicobasal localization and nuclear morphology (Fig. 3C,D). In the oversized retinae, the total cell number within the 30 µm wide column was increased by 15% on average in *rx2::caigf1r* retinae, with a more prominent increase in the ventral (21%) compared with the dorsal (10%) retina (Fig. 3E). The most prominent shift in the proportion of cell types was detected for ACs (24%) and BCs (31%) (Fig. 3F), while GCL and PRC numbers were rather similar in *rx2::caigf1r* and wild-type retinae (Fig. 3F). Some differences in cell type number changes were discernible between dorsal and ventral retina, with most distinctive increases in ACs, MG cells and BCs dorsally (Fig. S4A), and in ACs and BCs ventrally (Fig. S4B). These results indicate that Igf1r signaling activation in the CMZ shifts the neuroretinal cell type composition toward the INL and specifically to AC and BC fate.

### Igf1r signaling activation decreases cell cycle length in the CMZ

**Fig. 4.**
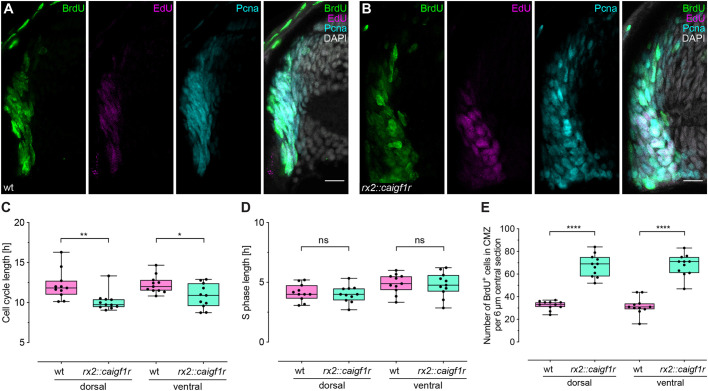
**Constitutive activation of Igf1r signaling decreases cell cycle length in the CMZ.** (A,B) Cryosections of wild-type (A) and *rx2::caigf1r* (B) hatchling retinae incubated for 2 h with BrdU and for 30 min with EdU to determine cell cycle length. BrdU (green), EdU (magenta) and Pcna (cyan) staining partially overlap in the CMZ. Scale bars: 10 μm. (C) Quantification of cell cycle length shows a reduction of 1-2 h in *rx2::caigf1r* (*n*=11 sections from four retinae in four fish) compared with wild-type (*n*=11 sections from four retinae in four fish) retinae (data obtained from two independent experiments; Mann–Whitney test, **P_d_=0.0018; *t*-test, *P_v_=0.0188). (D) Quantification of S-phase length in *rx2::caigf1r* (*n*=11 sections from four retinae in four fish) compared with wild-type (*n*=11 sections from four retinae in four fish) retinae (data obtained from two independent experiments; *t*-test, ^ns^*P*_d_=0.6764, ^ns^*P*_v_=0.8223). S-phase length is not altered in *rx2::caigf1r* retinae. (E) Quantification of BrdU-positive cell number in the CMZ per 6 μm central section shows that numbers have more than doubled in *rx2::caigf1r* (*n*=11 sections from four retinae in four fish) compared with wild-type (*n*=11 sections from four retinae in four fish) retinae (data obtained from two independent experiments; *t*-test, ****P_d/v_<0.0001). ns, not significant. Box plots show median, and 25th and 75th percentiles, with whiskers from minimum to maximum data points.

Igf1r signaling has been shown to influence cell cycle progression in different *in vitro* and *in vivo* models (Hodge et al., 2004; Schlueter et al., 2007). To determine the impact of the stem and progenitor cell-specific activation of Igf1r signaling in *rx2::caigf1r* on cell cycle duration, we next analyzed cell cycle and S-phase length of the proliferating cells in the CMZ of wild-type and *rx2::caigf1r* hatchlings. To this end, we employed a dual-pulse S-phase-labeling regime using BrdU and EdU (Das et al., 2009; Klimova and Kozmik, 2014). Hatchlings were incubated in BrdU for 2 h, washed and incubated in EdU for 30 min before analysis. BrdU, EdU and Pcna staining (Fig. 4A,B) allowed the quantification of the fractions of cells positive for one or more marker, allowing cell cycle and S-phase length to be determined. In the oversized *rx2::caigf1r* retinae, the cell cycle length was reduced from 12 h to 10.5 h on average (Fig. 4C), whereas the S-phase length remained constant with an average duration of 4.5 h (Fig. 4D). This had a drastic impact on the number of BrdU-positive cells in the CMZ, which was more than doubled in *rx2::caigf1r* compared with wild-type fish (Fig. 4E), a fact that is also confirmed by the analysis of the EdU- and Pcna-positive cells (Fig. S5A,B). The ratio of BrdU- to Pcna-positive cells increased from 50% in wild-type to 57% in *rx2::caigf1r* hatchlings in the dorsal CMZ, and from 55% to 60% in the ventral CMZ, indicating shortened G phases in the *rx2::caigf1r* CMZ.

Taken together, these data show that, in *rx2::caigf1r* hatchlings, more cells in the CMZ progress faster through the cell cycle, thereby expanding retinal cell number resulting in oversized retinae with shifted cell type proportions and increased height of the neuroretinal column.

### *Cndp* is a bona fide marker specific to neuroretinal stem cells

Differential responses of retinal stem and progenitor cells to extrinsic stimuli have been previously described (Centanin et al., 2014; Love et al., 2014). Given that an Igf signaling-mediated proliferative trigger in the combined stem and progenitor cell domain impacts on retinal size, we addressed the individual contribution of the stem and progenitor cell populations to the scaling response. This required to establish a retinal stem cell-specific driver as such a marker with the required specificity was not available for any vertebrate system. Exploiting existing resources for gene expression patterns, we focused on the cytosolic non-specific dipeptidase *zgc:114181* (hereafter *cndp*), which had been identified in a systematic *in situ* screen of full-length medaka cDNAs (Alonso-Barba et al., 2016) and showed a promising expression pattern at the retinal periphery.

**Fig. 5.**
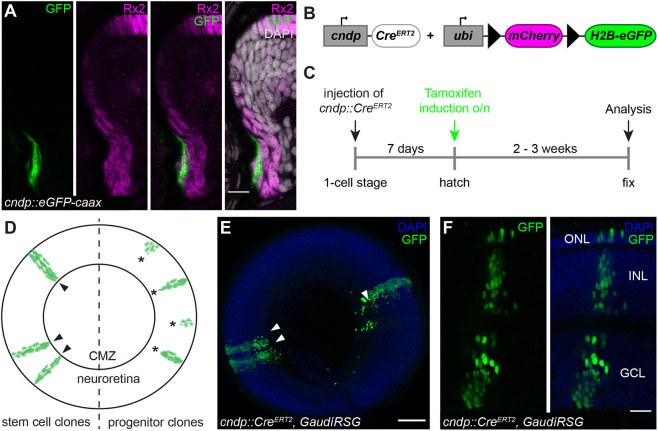
***Cndp* is expressed in multipotent neuroretinal stem cells.** (A) Cryosection of a *cndp::eGFP-caax* hatchling retina with GFP staining (green) in a peripheral subset of the Rx2 (magenta) domain in the CMZ. Scale bar: 10 μm. (B) Schematic representation of the constructs used for lineage tracing. Upon tamoxifen induction, *mCherry* is floxed out and *H2B-eGFP* is expressed in GaudíRSG fish. (C) Experimental outline. *cndp::Cre^ERT2^* is injected in one-cell stage GaudíRSG embryos. At hatching, fish are incubated in tamoxifen overnight and grown for 2-3 weeks before analysis. (D) Schematic representation of a whole-mount neuroretina containing stem cell clones (arrowheads) and progenitor clones (asterisks). Progenitor clones are not connected to the CMZ. (E,F) Whole-mount immunostaining of *cndp::Cre^ERT2^*, GaudíRSG retinae using GFP (green) with neuroretinal clones (E, arrowheads) labeling the whole retinal column (F), originating from multipotent neuroretinal stem cells (*n*=9 clones in four retinae from four fish, data obtained from two independent experiments). Scale bars: 100 μm in E; 20 μm in F.

To establish specific tools and address the expression of *cndp* relative to *rx2* we established transgenic reporter lines carrying putative regulatory regions driving a membrane-coupled GFP or nuclear mCherry reporter. The 5 kb genomic region upstream of the *cndp*-coding region was sufficient to recapitulate the mRNA expression pattern in transgenic reporter lines (*cndp::eGFP-caax* and *cndp::H2A-mCherry*) in the retina (Fig. 5A, Fig. S6A-D) as well as in the choroid plexi in the brain (Fig. S6A-D). In the retina, *cndp*-positive cells were found exclusively in a small, peripheral subset of the *rx2*-positive domain in the CMZ (Fig. 5A), with position likely representing retinal stem cells. To address the potency of those putative retinal stem cells and validate their nature (stem or progenitor cells), we employed a lineage analysis tool established in the lab that allows the determination of the potency of cells (Centanin et al., 2014). In brief, activation of Cre recombinase specifically expressed in *cndp*-positive cells was used to trigger a permanent switch in reporter gene expression in those cells and in all of their descendants. Stem cells and progenitor cells can be distinguished by their proliferative capacity. In the assay, stem cells generate continuous stripes (ArCoS) of labeled descending cells always connected to the CMZ. In contrast, progenitor cells, due to their limited proliferative capacity, generate footprints of labeled cells that are separated from the CMZ. We used the *cndp* promoter (Fig. 5B) characterized above to drive the expression of a tamoxifen-inducible Cre^ERT2^ and combined it with the switchable GaudíRSG reporter line (Centanin et al., 2014). A permanent switch of reporter expression from mCherry to GFP was triggered by the tamoxifen-induced activation of nuclear Cre activity. We characterized the distribution, continuity and cell type identity of the clonally labeled progeny to conclude the nature of the *cndp*-positive mother cell.

GaudíRSG embryos injected with the *cndp::Cre^ERT2^* plasmid at the one-cell stage were induced with tamoxifen at hatchling stage. After 2-3 weeks, fish were analyzed for GFP expression by whole-mount immunostaining (Fig. 5C). Retinae displayed GFP-positive clones that all originated in the peripheral CMZ and were continuous with the differentiated retina (Fig. 5D,E). This indicates that *cndp*-positive cells are neuroretinal stem cells. Clones comprised cells of all three nuclear layers (Fig. 5F), demonstrating multipotency of their cells of origin (Centanin et al., 2014). Importantly, we never observed clonal footprints originating from progenitor cells. This establishes *cndp* as a bona fide marker for neuroretinal stem cells and as an invaluable tool for dissecting stem versus progenitor cell behavior.

### Activation of Igf1r signaling in the CMZ preferentially increases progenitor cell numbers

**Fig. 6.**
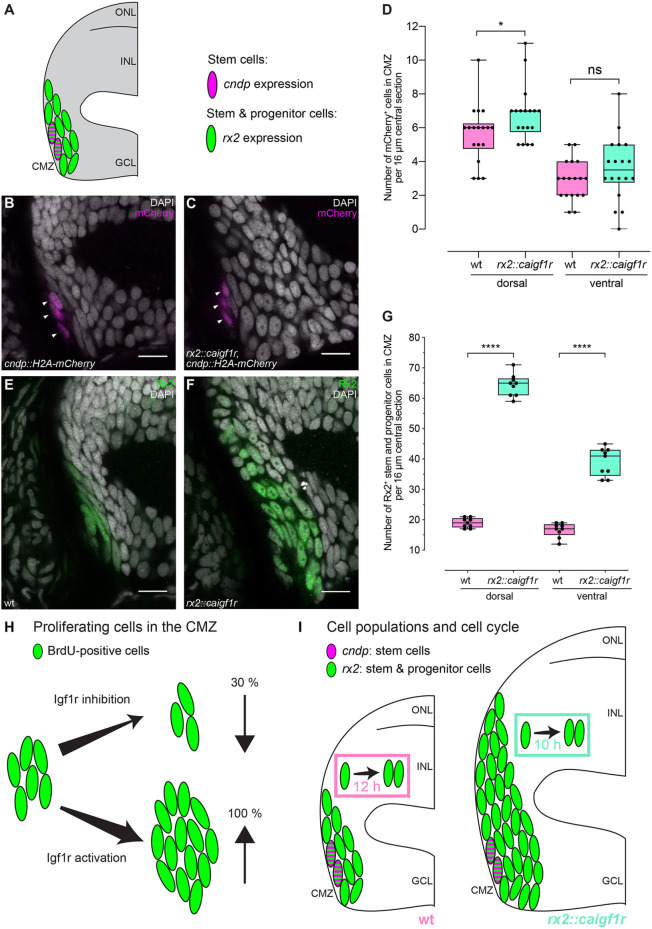
**Constitutive activation of Igf1r signaling expands retinal progenitor cell numbers.** (A) Schematic representation of the dorsal CMZ of a retinal section with *cndp* (magenta) and *rx2* (green) expression in stem and progenitor cells. (B,C) Cryosections of wild-type (B) and *rx2::caigf1r* (C) *cndp::H2A-mCherry* reporter hatchling retinae. mCherry (magenta) is visible in peripheral-most cells in the CMZ (arrowheads). (D) Quantification of H2A-mCherry-positive cell numbers in the CMZ per 16 µm central section does not indicate a major expansion of *cndp*-positive stem cells in *rx2::caigf1r* (*n*=18 sections from six retinae in three fish) compared with wild-type (*n*=18 sections from six retinae in three fish) retinae (Mann–Whitney test, *P_d_=0.0402; *t*-test, ^ns^*P*_v_=0.2177). (E,F) Cryosections of wild-type (E) and *rx2::caigf1r* (F) hatchling retinae. Rx2 staining (green) marks peripheral cells in the CMZ. (G) Quantification of Rx2-positive cell numbers in the CMZ per 16 µm central section demonstrates that Rx2-positive stem and progenitor cell numbers are more than doubled in *rx2::caigf1r* (*n*=9 sections from six retinae in three fish) compared with wild-type (*n*=9 sections from six retinae in three fish) retinae (*t*-test, ****P_d/v_<0.0001). (H) Igf1r inhibition decreases proliferating cells (green) in the CMZ by 30%, while Igf1r activation increases proliferation by at least 100%. (I) Igf1r activation in the CMZ expands progenitor numbers (*rx2*-positive, green) in *rx2::caigf1r* fish, but does not enlarge the stem cell population (*cndp*-positive, magenta). Cell cycle duration in the CMZ is shortened from 12 h in wild-type fish to 10 h upon Igf1r activation in *rx2::caigf1r* fish. ns, not significant. Box plots show median, and 25th and 75th percentiles, with whiskers from minimum to maximum data points. Scale bars: 10 μm.

To address whether stem or progenitor cell populations are expanded in response to activated Igf1r signaling, we took advantage of the stem cell-specific expression of *cndp* and used *rx2* as a marker for stem and progenitor cells (Fig. 6A). Retinal stem cells were identified by expressing the *cndp::H2A-mCherry* reporter in a stable transgenic line, and retinal stem and progenitor cells were identified by an antibody specific for Rx2 (Reinhardt et al., 2015). Numbers for retinal stem and progenitor cells were determined in hatchlings of wild type as well as in *rx2::caigf1r* retinae harboring the *cndp::H2A-mCherry* reporter (Fig. 6B,C).

Numbers of *cndp*-positive stem cells were comparable in wild-type and *rx2::caigf1r* retinae, ranging from 3 to 11 in the dorsal and from 0 to 8 stem cells per section in the ventral CMZ. The number of *cndp*-positive stem cells was rather stable, with a slight increase in *rx2::caigf1r* retinae (Fig. 6D). In contrast to that, the Rx2 domain was prominently expanded in *rx2::caigf1r* hatchlings (Fig. 6E,F), with Rx2-positive stem and progenitor cells in the CMZ more than doubled in *rx2::caigf1r* versus wild-type retinae (Fig. 6G), arguing that the progenitor, but not the stem cell, population is expanded by Igf1r signaling activation. In line with this observation, pIgf1r staining indicative of the activation of the Igf signaling cascade was detected only in progenitor cells and not in *cndp*-positive stem cells (Fig. S6E,F).

As retinal stem cell numbers in *rx2::caigf1r* hatchlings were almost comparable with wild type, we next wanted to understand whether retinal stem cells respond to the modulation of Igf1r signaling. We generated a transgenic line in which activation of Igf1r signaling was specifically targeted to *cndp*-positive retinal stem cells (*cndp::caigf1r*). Functionality of the construct was evident through enlarged choroid plexi in *cndp::caigf1r* fish (Fig. S7A,B). However, the GFP expression domain in the retina was unaltered (Fig. S7C,D). We examined relative eye size in hatchling and adult *cndp::caigf1r* fish, and neither stage displayed an alteration in relative eye size compared with wild-type siblings (Fig. S7E,F). These results demonstrate that the retinal stem cell population expressing *cndp* does not expand upon the activation of Igf1r signaling or by accumulation of *cndp* over time in adult stages. This further confirms and refines our findings that the progenitor, but not stem, cell population is expanded in *rx2::caigf1r* retinae.

Taken together, we demonstrate that retinal growth can be uncoupled from overall body growth through the activation of Igf1r signaling targeted to the CMZ. This intrinsic modulation elicits differential responses in stem and progenitor cell populations in the medaka retina, leading to a shortened cell cycle and consequential increase of retinal progenitors but not stem cell number (Fig. 6H,I).

## DISCUSSION

In this study, we have investigated how organ growth is coordinated with overall body growth in medaka by uncoupling growth control in the retina versus body. We focused on the retina and dissected the role of Igf1r signaling in regulating proliferation of stem cells versus progenitor cells in the retinal stem cell niche and its impact on retina size and composition at postembryonic stages. We show that proliferation in the retinal stem cell niche is dependent on and triggered by Igf1r-mediated mitogenic signaling. Combining expression analysis, inhibitor assays and gain-of-function approaches, we show that the Igf pathway is functioning in the postembryonic CMZ, where it actively controls proliferation. Our analyses indicate a localized paracrine signaling hub, with Igf1r activity in single progenitors in the CMZ. The inhibition of Igf1r signaling results in a decreased proliferation of CMZ cells. Conversely, the targeted constitutive activation of Igf signaling in the CMZ results in a clear increase of retina size and a shift in relative composition of the retinal layers. Strikingly, this is due to a specific response of the progenitor cells, while the proliferation of stem cells is not affected. Progenitor cells speed up their cell cycle without affecting the subsequent differentiation potential in response to Igf1r signaling activation (Fig. 6I). The specific activation of Igf signaling results in an increased proliferation rate in the progenitor pool that, in turn, causes a shift in retinal layer composition with the potential to impact on functional optical parameters.

Upon activation of Igf1r signaling in the CMZ, the thickness of the neuroretina increases by one-quarter. Intriguingly, the enlarged retinae are structurally intact, displaying correct lamination and differentiation. Although retinal enlargement due to enhanced progenitor proliferation might be an expected phenotype, the resulting intact retinal lamination with shifted cell type composition indicates a high degree of self-organization, depending on the number of progenitor cells. The specific action of the Igf/insulin pathway as signal that integrates nutritional status (Boulan et al., 2015) opens the field for interesting evolutionary hypotheses. In response to an Igf1r-mediated proliferative trigger in the progenitor cell domain, higher retinal columns, and thus an extended light path, are generated. Coordinated changes in relevant optical parameters, such as light path length, lens size and photoreceptor density, might therefore increase visual acuity. Thus, a signal representing nutrient availability can prompt an immediate selective advantage when preferentially acting on the retina and accessory tissues.

In the teleost retina, several populations of progenitor cells exist that differ in their proliferative and lineage capacities. Early progenitor cells in the CMZ are known to undergo self-renewing divisions generating two progenitor cells (PP), while old progenitor cells favor differentiating divisions (Wan et al., 2016). Among these are different lineage-specified progenitors that have been shown to produce different sets of cell type ratios upon modification of their transcriptional signatures (Pérez Saturnino et al., 2018). The activation of Igf1r signaling elicits two major changes in progenitor cells: first, increased numbers of Pcna- and rx2-positive cells indicates an increase in self-renewing PP divisions; second, a pronounced increase in ACs and BCs suggests that Igf1r activation might either convey certain cell fates or favor expansion of a specific progenitor population that is lineage committed to generate INL cells.

We showed that the uncoupling of Igf signaling in the retina by CMZ-specific constitutive activation of Igf1r signaling preferentially expands the *rx2*-positive progenitor but not the stem cell population. Targeting stem cells by specific Igf1r activation in *cndp*-expressing cells does not impact on retinal size, indicating that retinal stem cells do not respond to Igf1r signaling with an immediate and long-lasting increase in proliferation, nor could a cumulative effect be observed in adult fish. Conversely, progenitor cell numbers are more than doubled in response to constitutive activation of Igf1r signaling, rendering the *rx2*-positive progenitor population receptive for this mitogenic stimulus. Differential responses of stem and progenitor cells have been observed in the opposite direction after nutrient deprivation in the *Xenopus* retina. While stem cells are resistant to nutrient deprivation and to mTOR inhibition, retinal progenitors respond with changes in proliferation and differentiation in a mTOR-mediated manner (Love et al., 2014). These differences between retinal stem and progenitor cells are complementary to our observations whereby stem cells appear resistant to the Igf1r-mediated mitogenic stimulus, while progenitor proliferation is increased. Interestingly, outside the retina, divergence at the level of stem and progenitor cell behavior has been proposed as the basis for varying brain sizes in different amniote species (Nomura et al., 2013). Studies in geckos have proposed that progenitor cells are key for the evolutionary scaling of brain size in amniotes. The lower cell division rates of progenitor cells, their longer cell cycle and the predominance of self-renewing progenitor cell divisions are likely responsible for the comparatively smaller size of the gecko brain (Nomura et al., 2013).

Enlargement of the eye had been reported in few experimental and mutant conditions. Although the overexpression of *Yap* by mRNA injection could increase eye size in *Xenopus* embryos (Cabochette et al., 2015), retinal morphology and lamination were disturbed in those Yap-overexpressing tadpoles (Cabochette et al., 2015). Increase in retinal size has also been observed in a *patched2* mutant in zebrafish where retinal patterning and morphology are largely intact, but the number of MG cells is reduced (Bibliowicz and Gross, 2009). In the CMZ of *patched2* mutant embryos, the number of retinal progenitor cells is increased, but their cell cycle length is unaffected (Bibliowicz and Gross, 2009). We observed that, in response to active Igf1r signaling, the overall cell cycle was shortened without affecting S-phase length, in agreement with results from various systems in which Igf signaling influences G1- or G2-phase length (Hodge et al., 2004; Schlueter et al., 2007; Wang et al., 2015). The observed shortened cell cycle length of retinal progenitor cells of the oversized retinae is likely causal for the expansion of the CMZ and ultimately the entire retina in the *rx2::caigf1r* fish.

In contrast to eye size expansion resulting from increased retinal cell numbers, zebrafish and goldfish *bugeye* mutants exhibit enlarged eyes due to increased intraocular pressure (Kon et al., 2020; Stujenske et al., 2011; Veth et al., 2011). The *bugeye* phenotype is an adult-onset phenotype, and shows retinal cell density decrease and thinning of all nuclear layers. Moreover, *bugeye* mutants display relative refractive errors concordant with myopia due to vitreous chamber expansion between lens and retina (Veth et al., 2011), which displaces the retina behind the point at which the lens focuses light. Interestingly, the expansion of the eye in adult fish did not impact on the size of the lens in those mutants. We also did not observe an increase in lens size in *rx2::caigf1r* fish compared with wild-type siblings, indicating that there is no paracrine signal emanating from the neuroretinal niche in response to elevated Igf signaling to coordinate the size of the lens. Those data suggest an independent size control of retina and lens, as opposed to our previous results on neuroretina and retinal pigmented epithelium coordination, where behavior of neuroretinal stem cells in the CMZ drives retinal shape and impacts retinal pigmented epithelium stem cells that passively follow (Tsingos et al., 2019). Uncoupling neuroretinal Igf signaling from the rest of the body, as described here, provides an indication that independent control of retina and lens size exists during development and postembryonic growth. However, expression of the insulin receptor and IGF1 receptor at high levels at the lens periphery in embryonic and P0 mice (Xie et al., 2007), as well as proliferation of lens epithelial cells upon IGF1 treatment in rat lens epithelial explants (Iyengar et al., 2006), indicates that control of lens growth and size could also be modulated by Igf signaling. In the future, it will be important to address whether Igf signaling impacts lens germinal cell proliferation in the medaka retina and whether Igf signaling contributes to the regulation of lens and retina sizes to achieve a continuous functional consistency during the life-long growth of the eye.

The targeted isolated size increase of the retina and the concomitant change in cell type composition opens a wide area for discussing the evolutionary and ecological significance of coordinating retinal size and architecture. Throughout the teleost clade, adaptations to specific habitats and niches are evident in the retinal architecture. Surface-dwelling fish, such as medaka, have two layers of PRCs, one light-sensitive rod and one cone layer responsible for color vision, while zebrafish possess one layer of cones as well as three to four layers of rods for enhanced light perception, as they live in deeper waters (Lust and Wittbrodt, 2018). One particularly interesting example of retinal architecture changes is the ‘four-eyed’ fish *Anableps anableps*, which displays structural differences within the dorsal and ventral retina to accommodate its specific optic requirements. The ventral retina features an INL that is twice as thick as the dorsal INL, concordant with increased proliferation in the ventral compared with the dorsal CMZ during the development of the larval eye (Perez et al., 2017). As we observe a preferential increase in the INL neuron population in *rx2::caigf1r*, it is tempting to speculate that an altered proliferation of progenitor cells (initiated by altered Igf signaling) in the ventral CMZ contributes to manifesting the structural differences in the *Anableps anableps* retina. We speculate that Igf signaling can act as an evolutionary handle through which retinal size, morphology, cell type composition and, consequently, function can be adapted by modifying signaling activity in distinct populations of progenitor cells in the CMZ and beyond.

## MATERIALS AND METHODS

### Animals and transgenic lines

Medaka (*Oryzias latipes*) used in this study were kept as closed stocks at Heidelberg University. All experimental procedures and husbandry were performed in accordance with the German animal welfare law and approved by the local government (Tierschutzgesetz §11, Abs. 1, Nr. 1, husbandry permit AZ 35–9185.64/BH and line generation permit AZ 35–9185.81/G-145-15). Fish were maintained in a constant recirculating system at 28°C on a 14 h light/10 h dark cycle. The following stocks and transgenic lines were used: wild-type Cabs, *Heino* mutants (Loosli et al., 2000), *rx2::caigf1r rx2::lifeact-eGFP*, *cndp::H2A-mCherry*, *cndp::eGFP-caax*, *cndp::H2B-eGFP*, *cndp::caigf1r cndp::H2B-eGFP*, *cndp::Cre^ERT2^* and *GaudíRSG* (Centanin et al., 2014). All transgenic lines were created by microinjection with Meganuclease (I-SceI) in medaka embryos at the one-cell stage, as previously described (Thermes et al., 2002).

The constitutively active Igf1r variant (Cd8a:Igf1ra, caIgf1r) was generated by an in-frame fusion of the codon-optimized extracellular and transmembrane domain of olCd8a (synthesized by Geneart) and the intracellular domain of the medaka Igf1ra, as previously described (Carboni et al., 2005). To generate the *rx2::caigf1r rx2::lifeact-eGFP* line, two F0 fish were crossed, and each subsequent generation was derived from one transgenic male outcrossed to wild-type Cabs. All experiments were carried out in the F2 to F4 generations.

The *cndp::Cre^ERT2^* plasmid (*cndp* Ensembl ID: ENSORLG00000003701) was generated by cloning the 5 kb *cndp* regulatory region in a pBS/I-SceI-vector containing a tamoxifen-inducible Cre recombinase. The plasmid contains *cmlc2::eCFP* as insertional reporter.

### BrdU/EdU incorporation

For BrdU incorporation, hatchlings (stage 40; Iwamatsu, 2004) were incubated in 2.5 mM BrdU (Sigma-Aldrich) diluted in 1×embryo rearing medium (ERM; 17 mM NaCl, 40 mM KCl, 0.27 mM CaCl_2_, 0.66 mM MgSO_4_ and 17 mM HEPES) for 2 h. For EdU incorporation, hatchlings were incubated for 30 min in 250 µM EdU (ThermoFisher) diluted in 1×ERM. Quantification of BrdU-positive cells was performed in four retinae from individual hatchlings. Cell counts were performed in z=6 µm of two or three central sections per retina.

### Igf1r inhibition

For inhibition of Igf1r, hatchling fish were incubated in 10 μM NVP-AEW541 (Selleckchem, 10 mM stock solution solved in DMSO) diluted in 1×ERM at 28°C for 24 h. In a parallel control group, hatchling fish were incubated in 0.1% DMSO/1×ERM at 28°C for 24 h. Directly afterwards, fish were euthanized and fixed for analysis.

### Induction of Cre/lox system

For Cre^ERT2^ induction, hatchlings were treated with a 5 µM tamoxifen solution (Sigma-Aldrich) in 1×ERM overnight.

### Immunohistochemistry on cryosections

Fish were euthanized using 20× Tricaine and fixed overnight in 4% PFA, 1× PTW at 4°C. After fixation, samples were washed with 1× PTW and cryoprotected in 30% sucrose in 1× PTW at 4°C. To improve section quality, the sections were incubated in a half/half mixture of 30% sucrose and Tissue Freezing Medium (Leica) for at least 3 days at 4°C. Serial sections (16 µm) were obtained on a cryostat. Sections were rehydrated in 1× PTW for 30 min at room temperature. Blocking was performed for 1-2 h with 10% NGS (normal goat serum) in 1× PTW at room temperature. The respective primary antibodies were applied diluted in 1% NGS overnight at 4°C. The secondary antibody was applied in 1% NGS together with DAPI (Sigma-Aldrich, D9564; 1:500 dilution in 1× PTW of 5 mg/ml stock) for 2-3 h at 37°C. Slides were mounted with 60% glycerol and kept at 4°C until imaging.

### BrdU and Pcna immunohistochemistry on cryosections

BrdU and Pcna antibody staining was performed with an antigen retrieval step. After all antibody staining and DAPI staining, except for BrdU/Pcna, were complete, a fixation for 30 min was performed with 4% PFA. Slides were incubated for 1.5 h at 37°C in 2 N HCl solution, and pH was recovered by washing with a 40% Borax solution in 1× PTW before incubation with the primary BrdU or Pcna antibody.

### EdU staining on cryosections

The EdU staining reaction was performed after all other antibody staining was completed using the Click-iT EdU Alexa Fluor 647 Flow Cytometry Assay Kit according to the manufacturer's protocol (Thermo Fisher).

### TUNEL staining on cryosections

TUNEL staining was performed after all other antibody staining was completed using the In Situ Cell Death Detection Kit Fluorescein (Roche). Staining was performed according to the manufacturer's protocol with the following modification: washes were performed with 1× PTW instead of PBS.

### Immunohistochemistry on whole-mount retinae

Fish were euthanized using 20× Tricaine and fixed overnight in 4% PFA in 1× PTW at 4°C. After fixation, samples were washed with 1× PTW. Fish were bleached with 3% H_2_O_2_, 0.5% KOH in 1× PTW for 2-3 h in the dark. Retinae were enucleated and permeabilized with acetone for 15 min at −20°C. Blocking was performed in 1% bovine serum albumin (Sigma-Aldrich), 1% DMSO (Roth/Merck), 4% sheep serum (Sigma-Aldrich) in 1× PTW for 2 h. Samples were incubated with primary antibody in blocking buffer overnight at 4°C. The secondary antibody was applied together with DAPI in blocking buffer overnight at 4°C. Primary antibodies were used at 1:200, secondary antibodies at 1:250 and DAPI at 1:500.

### Antibodies

The following primary antibodies were used: anti-BrdU (rat; Abcam, ab6326; 1:200), anti-DsRed (rabbit; Clontech, 632496; 1:500), anti-eGFP (chicken; Life Technologies, A10262; 1:500), anti-GS (mouse; Chemicon, MAB302; 1:500), anti-Otx2 (goat; R&D systems, AF1979; 1:200), anti-pAkt (rabbit; Cell Signaling, 4060; 1:200), anti-Pcna (mouse; Millipore, CBL407; 1:100), anti-pIgf1r (rabbit; Abcam, ab39398; 1:100), anti-Rx2 (rabbit; Reinhardt et al., 2015; 1:500) and anti-Zpr-1 (mouse; ZIRC, ZDB-ATB-081002-43; 1:200). The following secondary antibodies were used (at 1:750): anti-chicken Alexa Fluor 488 (donkey; Jackson, 703-485-155), anti-goat Alexa 633 (donkey; Life Technologies, A-21082), anti-mouse Alexa 546 (goat; Life Technologies, A-11030), anti-mouse Alexa 647 (donkey; Jackson, 715-605-151), anti-rabbit Alexa Fluor 488 (goat; Life Technologies, A-11034), anti-rabbit DyLight549 (goat; Jackson, 112-505-144), anti-rabbit Alexa Fluor 647 (goat; Life Technologies, A-21245) and anti-rat DyLight488 (goat; Jackson, 112-485-143). DAPI (Sigma-Aldrich, D9564) nuclear counterstaining was performed as described by Inoue and Wittbrodt (2011).

### Measurement of cell cycle and S phase length

To determine cell cycle length of retinal progenitor cells, dual-pulse S-phase labeling with BrdU and EdU were used as previously described (Das et al., 2009; Klimova and Kozmik, 2014). This approach allows an estimation of S-phase length by consecutive BrdU and EdU incubation, and subsequent extrapolation of total cell cycle length in the Pcna-positive progenitor population. Hatchlings were incubated for 2 h in BrdU, then 30 min in EdU before fixation. Pcna antibody staining was used to label all cycling retinal progenitor cells. Pcna-, EdU- and BrdU-positive cells, as well as cells positive for only BrdU, were quantified.

Cell-cycle length and S-phase length were determined with the following formulas: T_cell cycle_=2 h *(Pcna^+^ cells/BrdU^+^ EdU^−^) as calculation of the ratio of all cycling cells (Pcna^+^) to S phase exit (BrdU^+^ EdU^−^); T_S phase_=2 h *(EdU^+^ cells/BrdU^+^ EdU^−^) as calculation of the ratio of S-phase entry (EdU^+^) to S-phase exit (BrdU^+^ EdU^−^).

Four retinae from individual hatchlings were used for analysis. Cell counts were performed in z=6 µm of two or three central sections per retina. T_cell cycle_ and T_S phase_ were determined for individual sections.

### Whole-mount *in situ* hybridization

Whole-mount *in situ* hybridizations using NBT/BCIP detection were carried out as previously described (Loosli et al., 1998). Afterwards, samples were cryoprotected in 30% sucrose in 1× PTW overnight at 4°C and 20 µm thick serial sections were obtained on a Leica cryostat. Sections were rehydrated in 1× PTW for 30 min at room temperature and washed several times with 1× PTW. Slides were mounted with 60% glycerol and kept at 4°C until imaging.

### Image acquisition

All immunohistochemistry images were acquired by confocal microscopy at a Leica TCS SP8 with 20× or 63× glycerol objective. Sections of whole-mount *in situ* hybridization were imaged using a Zeiss Axio Imager M1 microscope. Images of whole hatchlings were acquired with a Nikon SMZ18 stereomicroscope equipped with aNikon DS-Ri1 camera.

### Image processing and statistical analysis

Images were processed via Fiji image processing software. Statistical analysis and graphical representation of the data were performed using the Prism software package (GraphPad). Box plots show median, and 25th and 75th percentiles, with whiskers from minimum to maximum data points. All data points, represented by black dots, are superimposed on box plots. Normal distribution was tested with the Shapiro-Wilk normality test, and unpaired two-tailed *t*-tests or Mann–Whitney tests were performed to determine statistical significance. The *P*<0.05 was considered significant, *P* values are given in the figure legends. Sample size (*n*) is mentioned in every figure legend, all sections were treated as independent samples. No statistical methods were used to predetermine sample sizes, but our sample sizes are similar to those generally used in the field. The experimental groups were allocated randomly; no blinding was carried out during allocation.

## Supplementary Material

Supplementary information

Reviewer comments
