# Supplementary material for: Igf signaling couples retina growth with body growth by modulating progenitor cell division
Source: Development. 2021 Apr 15;148(7):dev199133. doi: 10.1242/dev.199133 (PMC8077508; doi:10.1242/dev.199133)
Supplement: Supplementary information [file develop-148-199133-s1.pdf]

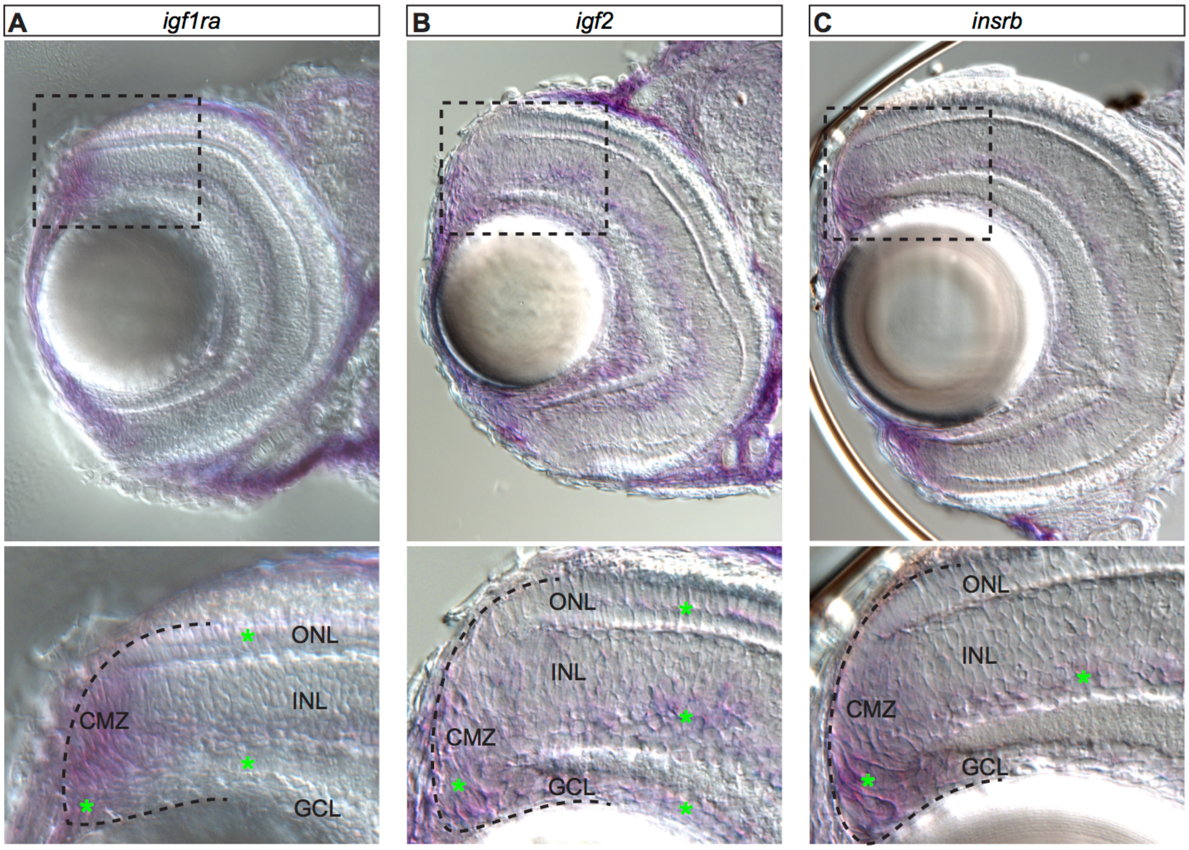

Fig. S1: Igf signaling pathway components are expressed in the hatchling CMZ. (A-C) Cryosections of whole-mount *in situ* hybridizations of hatchling retinae (n = 3 fish each). Expression of *igf1ra* (A) is visible in CMZ, outer (ONL) and inner (INL) nuclear layer (asterisks). *Igf2* (B) is expressed in CMZ, ONL, INL and ganglion cell layer (GCL). CMZ and INL show expression of *insrb* (C).

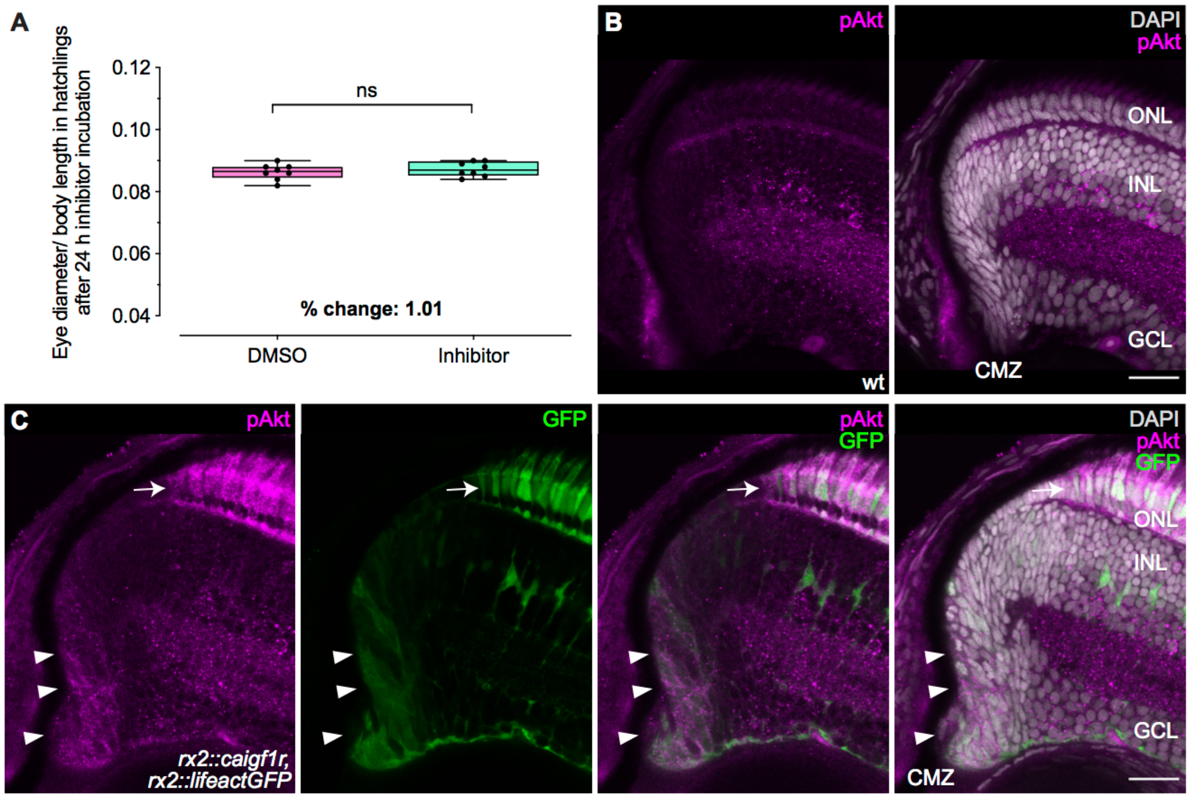

Fig. S2: *Caigf1r* expression results in increased downstream signaling activation in the CMZ. (A) Quantification of relative eye size (eye diameter normalized to body length) of wt hatchlings incubated for 24 h in DMSO (n = 8) and 10 μM Igf1r inhibitor NVP-AEW541 (n = 8) (t-test: $^{ns}P = 0.4799$). The percentage change of relative eye size means of DMSO- and inhibitor-treated hatchlings is 1.01%. (B,C) Cryosections of wt (B) and *rx2::caigf1r* (C) retinae at hatching stage. The pAkt-positive domain (magenta, arrowheads) is enlarged in *rx2::caigf1r* (C) compared to wt (B) retinae, co-localizing with GFP signal (green) also in PRCs (C, arrow) (n = 3 fish each). Scale bars are 10 μm. ns, not significant. Box plots depict median + quartiles with min-max whiskers.

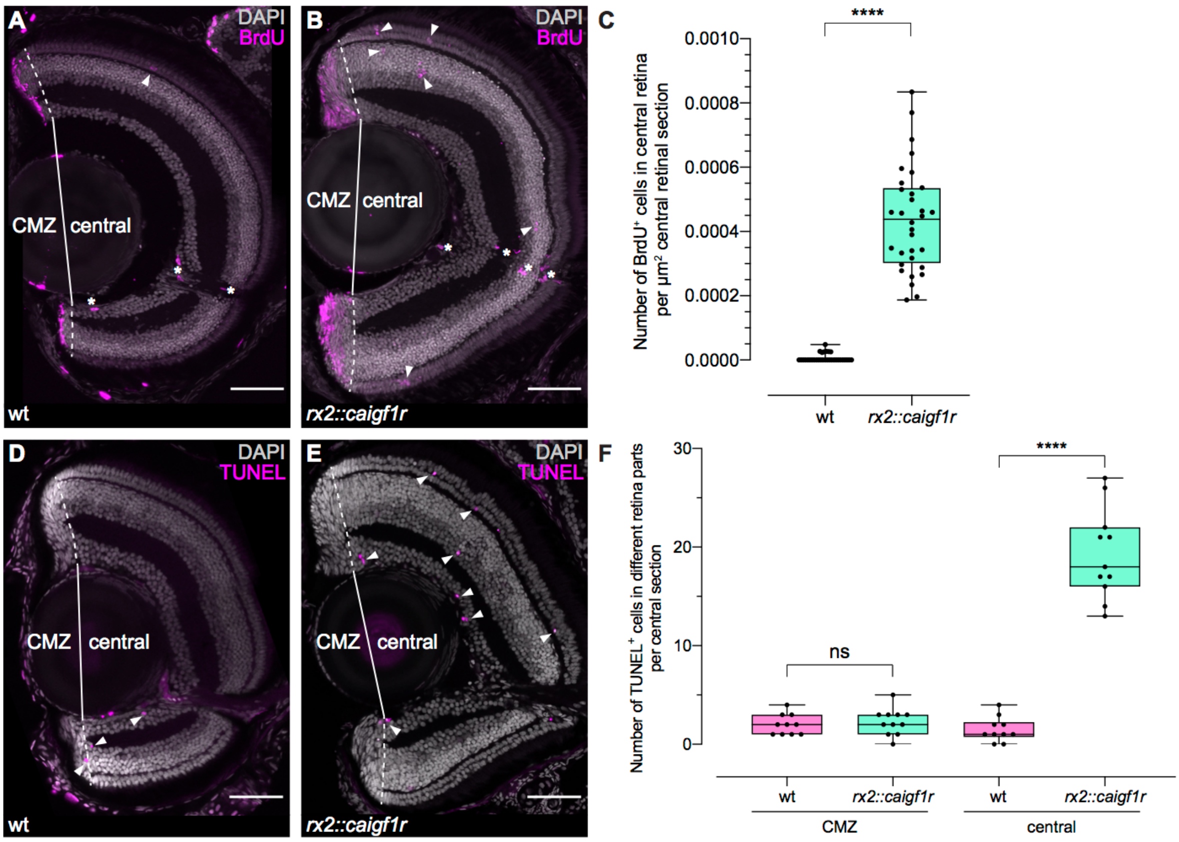

Fig. S3: Neuroretinal expansion in *rx2::caigf1r* retinae is driven neither by central retina proliferation nor by decreased apoptosis. (A,B) Cryosections of wt (A) and *rx2::caigf1r* (B) retinae at hatching stage fixed after 2h BrdU incubation. BrdU-positive cells (magenta, arrowheads) are present in the central region of wt (A) and *rx2::caigf1r* (B) retinae. Asterisks mark BrdU-positive cells in the lens epithelium and the optic nerve. Scale bars are 50 µm. (C) Quantification of BrdU-positive cell number in the central retina per 16 µm section normalized to retinal area [μm^2] shows slightly elevated, very low numbers in *rx2::caigf1r* (n = 32 sections from 8 retinae in 4 fish) compared to wt (n = 32 sections from 8 retinae in 4 fish) retinae (Mann-Whitney test: ****P > 0.0001). (D,E) Cryosections of wt (D) and *rx2::caigf1r* (E) retinae at hatching stage. TUNEL-positive nuclei (magenta, arrowheads) are present in *rx2::caigf1r* (D) and wt (E) retinae. Scale bars are 50 µm. (F) Quantification of TUNEL-positive cell number per 16 µm section shows increased apoptosis in the central but not CMZ region of *rx2::caigf1r* (n = 11 sections from 8 retinae in 4 fish) compared to wt (n = 10 sections from 8 retinae in 4 fish) retinae (t-test: $^{ns}P_{CMZ}$ = 0.6142, $^{****}P_{central}$ > 0.0001). ns, not significant. Box plots depict median + quartiles with min-max whiskers.

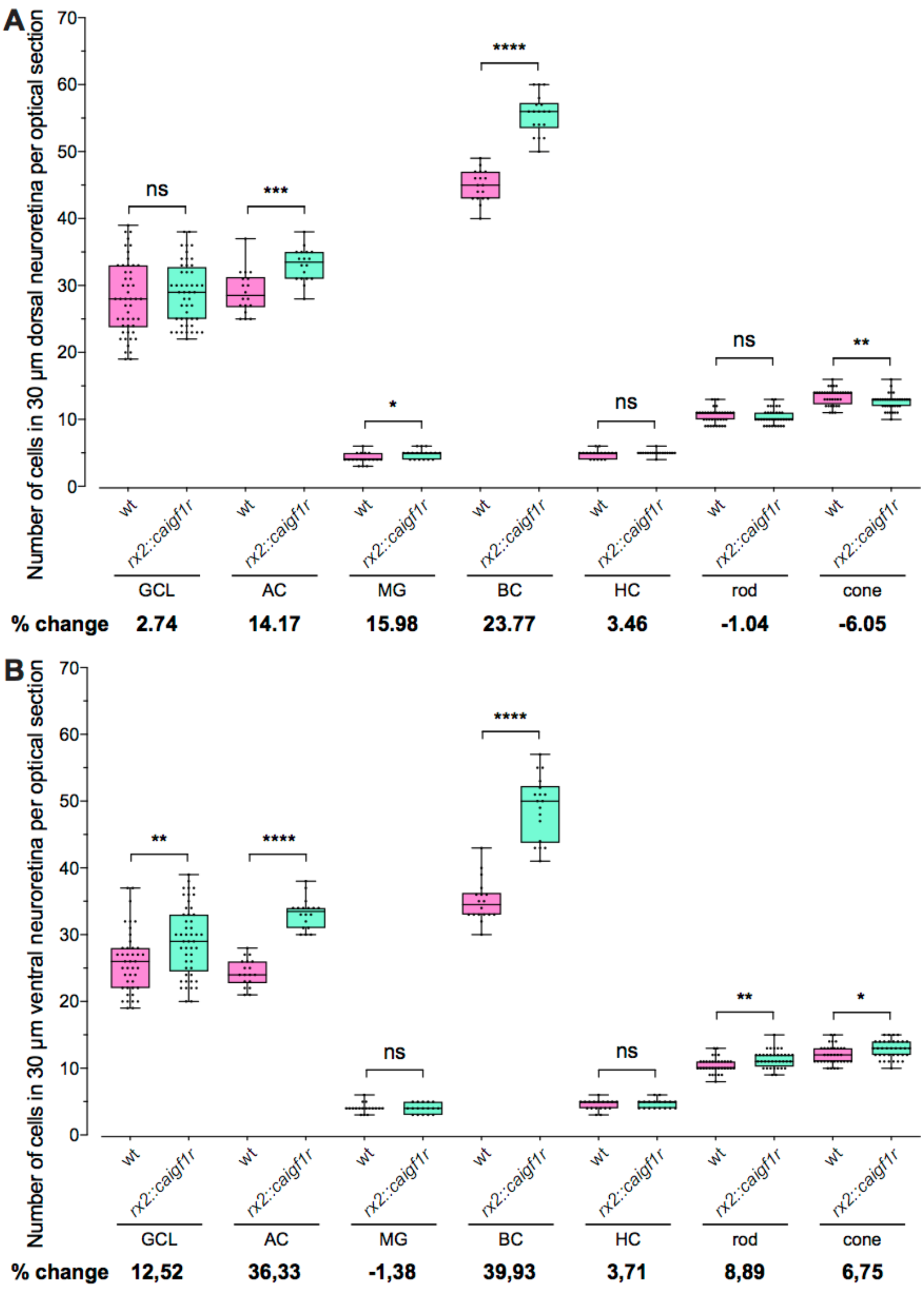

Fig. S4: Cell type composition of the dorsal and ventral neuroretina is changed in *rx2::caigf1r* fish. (A) Quantification of cell type number in a 30 µm wide region in the dorsal differentiated peripheral neuroretina shows increased AC and BC number in *rx2::caigf1r* (n ≥ 35 sections from 6 retinae in 3 fish) compared to wt (n ≥ 36 sections from 6 retinae in 3 fish) retinae (t-test: $^{***}P_{AC} = 0.0001$, $^{***}P_{BC} < 0.0001$, Mann-Whitney test: $^{ns}P_{GCL} = 0.4193$, $^{*}P_{MG} = 0.0127$, $^{ns}P_{HC} = 0.2326$, $^{ns}P_{rod} = 0.6162$, $^{**}P_{cone} = 0.0031$). Percentage change of the cell number means of wt and *rx2::caigf1r* shows large changes (≥ 10%) in AC, MG and BC number. (B) Quantification of cell type number in

Development • Supplementary information

a 30 µm wide region in the ventral differentiated peripheral neuroretina shows increased GCL, AC and BC number in *rx2::caigf1r* (n ≥ 35 sections from 6 retinae in 3 fish) compared to wt (n ≥ 36 sections from 6 retinae in 3 fish) retinae (t-test: $^{**}P_{GCL}$ = 0.0023, $^{****}P_{AC}$ < 0.0001, $^{****}P_{BC}$ < 0.0001, Mann-Whitney test: $^{ns}P_{MG}$ = 0.8407, $^{ns}P_{HC}$ = 0.6608, $^{**}P_{rod}$ = 0.0017, $^{*}P_{cone}$ = 0.0150). Percentage change of the cell number means of wt and *rx2::caigf1r* shows large changes (≥ 10%) in GCL, AC and BC number. ns, not significant. Box plots depict median + quartiles with min-max whiskers.

Development • Supplementary information

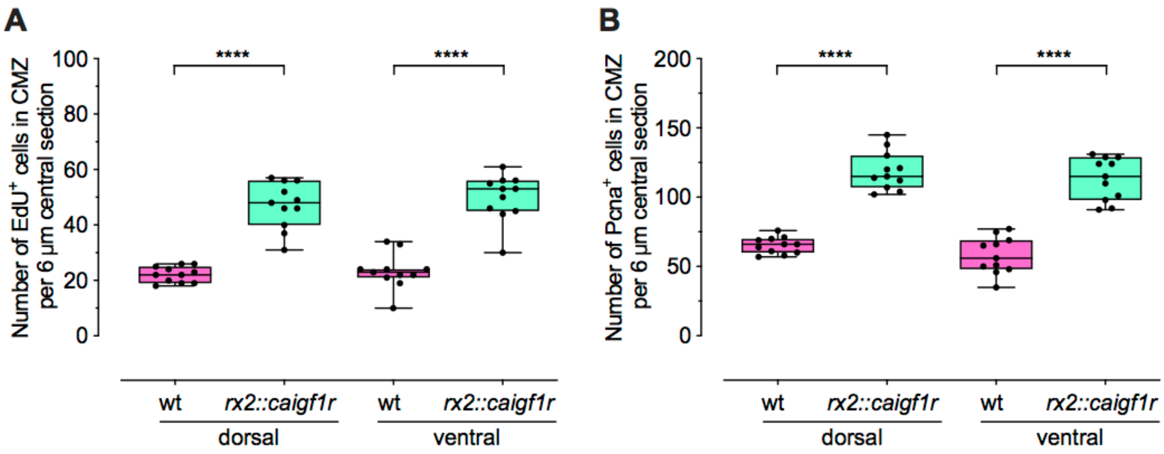

Fig. S5: EdU- and Pcna-positive cell number is increased in *rx2::caigf1r* retinae. (A,B) Quantification of EdU- (A) and Pcna-positive (B) cell number in the CMZ per 6 µm central section shows increased numbers in *rx2::caigf1r* (n = 11 sections from 4 retinae in 4 fish) compared to wt (n = 11 sections from 4 retinae in 4 fish) retinae (data obtained from two independent experiments; t-test: ****$P_{d/v} < 0.0001$). Box plots depict median + quartiles with min-max whiskers.

Development • Supplementary information

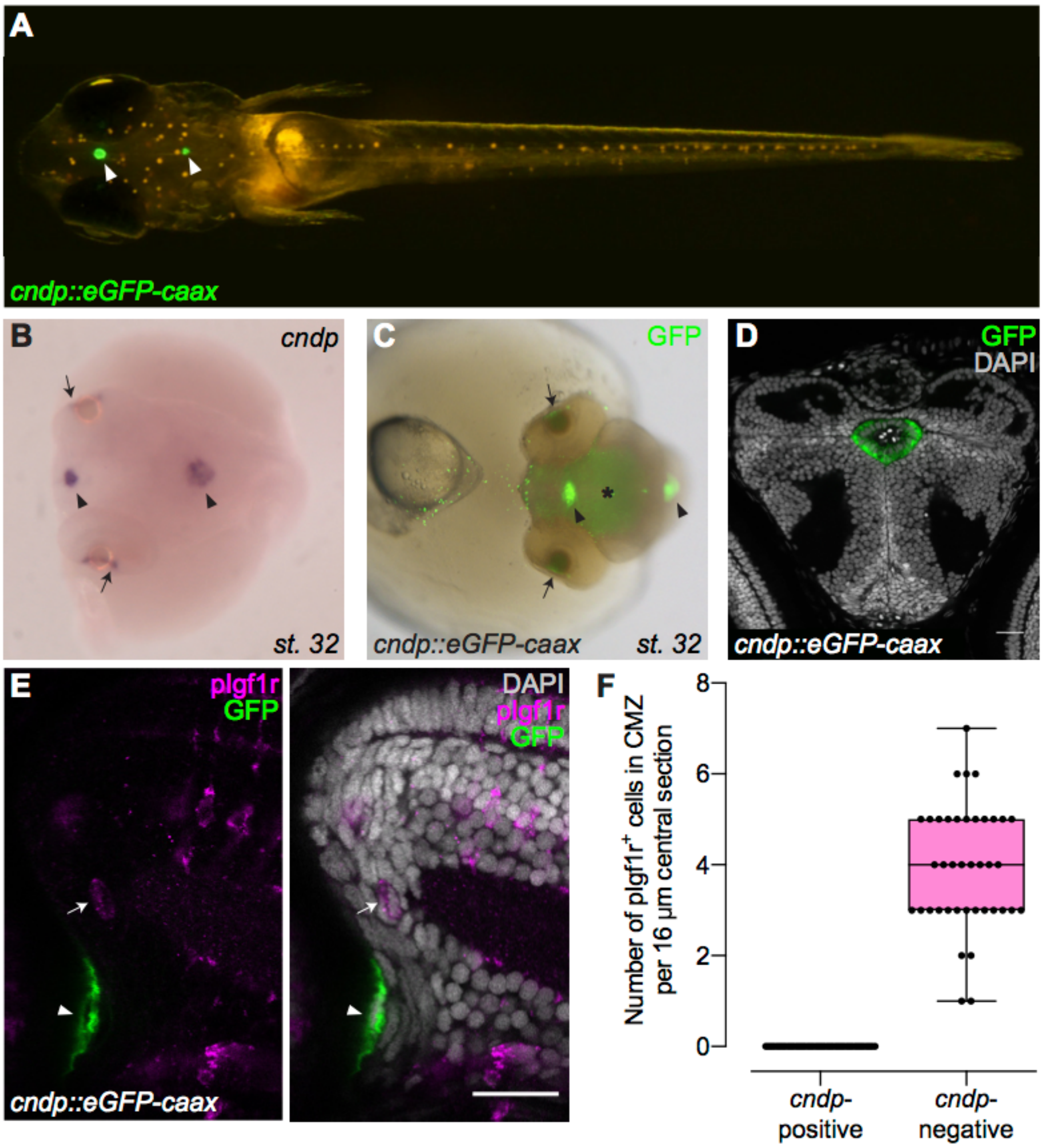

Fig. S6: *Cndp* is expressed in the choroid plexi. (A) *Cndp::eGFP-caax* hatchling shows GFP expression in the choroid plexi in the brain (arrowheads). Retinal GFP expression is masked by the retinal pigmented epithelium. (B) Whole-mount *in situ* hybridization for cndp in a stage 32 medaka embryo (dorsal view). Expression of *cndp* is visible in the peripheral retina (arrows) and the choroid plexi (arrowheads). Adapted from MEPD/(Alonso-Barba et al., 2016). (C) Whole-mount immunostaining against GFP (green) in a stage 32 *cndp::eGFP-caax* embryo. GFP expression (green) is visible in the peripheral retina (arrows) and the choroid plexi (arrowheads). Asterisk indicates unspecific background staining. (D) Cryosection of a *cndp::eGFP-caax* hatchling brain. The diencephalic choroid plexus is positive for GFP (green). (E) Cryosection of *cndp::eGFP-caax* hatchling with anti-pIgf1r (magenta) staining shows Igf1r activity in single progenitors (arrow) but not in *cndp*-positive stem cells (green,

Development • Supplementary information

arrowhead). Scale bars are 20 µm. (F) Quantification of pIgf1r-positive/*cndp*-negative and pIgf1r/*cndp*-double positive cell numbers in the CMZ per 16 µm section show no GFP-positive cells co-labeled with pIgf1r (n = 157 cells in 40 sections from 5 fish). Box plots depict median + quartiles with min-max whiskers.

Development • Supplementary information

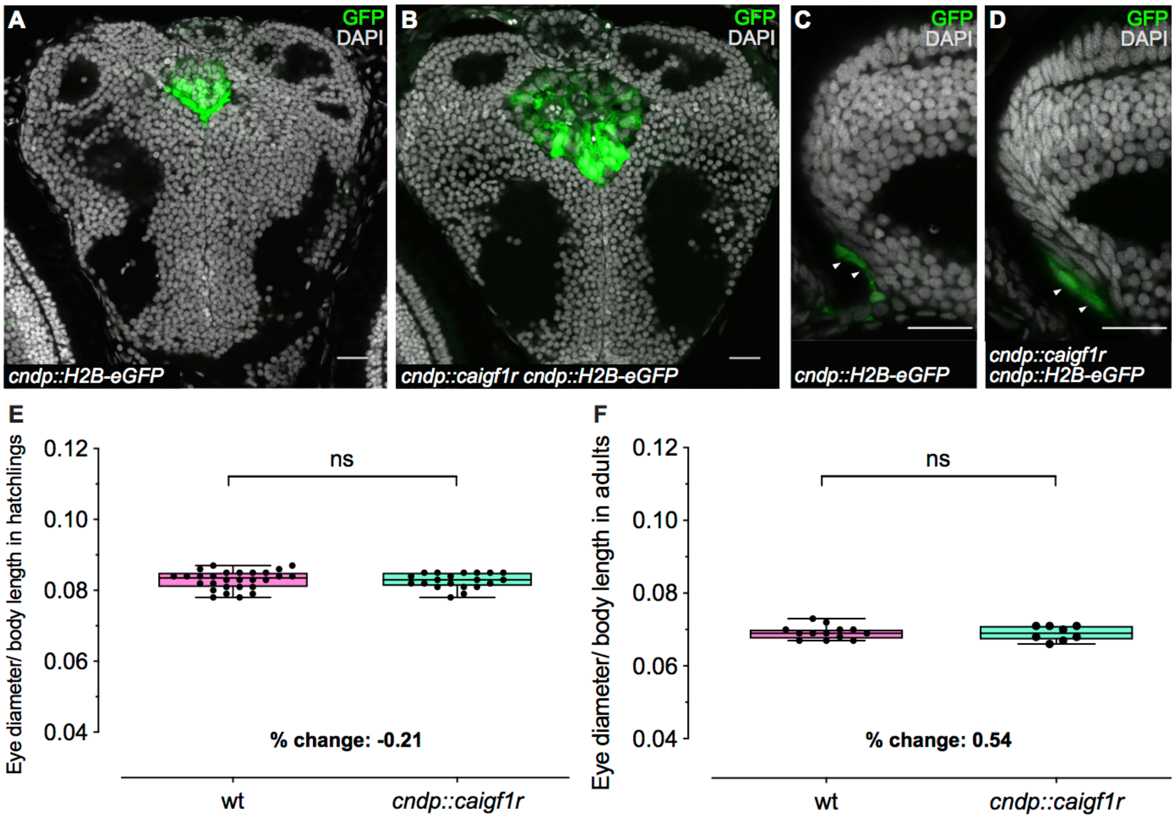

Fig. S7: Expression of *caigf1r* in retinal stem cells does not result in increased eye size. (A-D) Cryosections of wt (A,C) and *cndp::caigf1r* (B,D) *cndp::H2B-eGFP* reporter hatchlings. The GFP-positive (green) choroid plexi are enlarged in *cndp::caigf1r* (B) compared to wt (A) brains. *Cndp*-driven GFP expression (green, arrowheads) in the CMZ of *cndp::caigf1r* hatchlings (C) is not expanded compared to wt (D) (n = 3 fish each). Scale bars are 20 µm. (E) Quantification of relative eye size (eye diameter normalized to body length) of wt (n = 28) and *cndp::caigf1r* (n = 20) hatchlings (Mann-Whitney test: $^{ns}P = 0.7023$). (F) Quantification of relative eye size (eye diameter normalized to body length) of wt (n = 8) and *cndp::caigf1r* (n = 13) 3-month-old adult fish (t-test: $^{ns}P = 0.7894$). The percentage change of relative eye size means of wt and *cndp::caigf1r* is > 1% in hatchlings (E) and adults (F). ns, not significant. Box plots depict median + quartiles with min-max whiskers.

Table S1. Raw data and calculated values for relative eye size quantification at hatchling stage, with eye diameter measured in the anterior-posterior axis

Click here to download Table S1

Table S2: Raw data and calculated values for relative eye size quantification at hatchling stage, with eye diameter measured in the anterior-posterior axis.

Genotype	Fish	eye diameter [a.u.]	body length [a.u.]	ratio eye diameter/ body length
wildtype	1	234'173	3'546'325	0.066
wildtype	2	238'212	3'405'529	0.070
wildtype	3	225'320	3'378'161	0.067
wildtype	4	238'212	3'420'000	0.070
wildtype	5	230'083	3'370'928	0.068
rx2::caigf1r	1	228'886	3'157'283	0.072
rx2::caigf1r	2	251'034	3'285'564	0.076
rx2::caigf1r	3	228'729	2'946'055	0.078
rx2::caigf1r	4	201'022	2'759'481	0.073
rx2::caigf1r	5	243'167	3'231'273	0.075
rx2::caigf1r	6	235'342	3'309'665	0.071
rx2::caigf1r	7	224'860	3'022'252	0.074
wildtype			median	0.068
			standard deviation	0.0017
rx2::caigf1r			median	0.074
			standard deviation	0.0023

Development • Supplementary information
