# Supplementary material for: Igf signaling couples retina growth with body growth by modulating progenitor cell division
Source: Development. 2021 Apr 15;148(7):dev199133. doi: 10.1242/dev.199133 (PMC8077508; doi:10.1242/dev.199133)
Supplement: Reviewer comments [file develop-148-199133_review_history.pdf]

Igf signaling couples retina growth with body growth by modulating progenitor cell division

Clara Becker, Katharina Lust and Joachim Wittbrodt
DOI: 10.1242/dev.199133

Editor: Steve Wilson

Review timeline
Original submission: 2 December 2020
Editorial decision: 11 January 2021
First revision received: 2 February 2021
Editorial decision: 23 February 2021
Second revision received: 24 February 2021
Accepted: 4 March 2021

Original submission

<u>First decision letter</u>

MS ID#: DEVELOP/2020/199133

MS TITLE: Igf signaling coordinates retina growth with body growth by modulating progenitor cell division

AUTHORS: Clara Becker, Katharina Lust, and Joachim Wittbrodt

A happy new year to you. I have now received all the referees' reports on the above manuscript, and have reached a decision. The referees' comments are appended below, or you can access them online: please go to BenchPress and click on the 'Manuscripts with Decisions' queue in the Author Area.

As you will see, the referees express considerable interest in your work, but have some criticisms and suggestions for improvements to your manuscript. If you are able to revise the manuscript along the lines suggested, I will be happy receive a revised version of the manuscript. Please also note that Development will normally permit only one round of major revision.

We are aware that you may be experiencing disruption to the normal running of your lab that make experimental revisions challenging. If it would be helpful, we encourage you to contact us to discuss your revision in greater detail. Please send us a point-by-point response indicating where you are able to address concerns raised (either experimentally or by changes to the text) and where you will not be able to do so within the normal timeframe of a revision. We will then provide further guidance. Please also note that we are happy to extend revision timeframes as necessary.

Please attend to all of the reviewers' comments and ensure that you clearly highlight all changes made in the revised manuscript. Please avoid using 'Tracked changes' in Word files as these are lost in PDF conversion. I should be grateful if you would also provide a point-by-point response detailing how you have dealt with the points raised by the reviewers in the 'Response to Reviewers' box. If you do not agree with any of their criticisms or suggestions please explain clearly why this is so.

© 2021. Published by The Company of Biologists under the terms of the Creative Commons Attribution License (https://creativecommons.org/licenses/by/4.0/).

Reviewer 1

Advance summary and potential significance to field

In this paper, Becker et al. investigate the role of Igf signalling in the regulation of tissue size, more specifically in the medaka fish retina. They find that inhibition of Igf1r-mediated signalling using a small molecule inhibitor leads to reduced proliferation in the CMZ. Conversely, targeted activation of Igf1r signalling in the CMZ increases proliferation, which leads to increased retina and eye size, without affecting the overall size of the fish. Interestingly, the larger retinas observed following activation of Igf1r in the CMZ contain more retinal ganglion cells, amacrine cells, and bipolar cells, but no change in other cell types. The authors further show that the increased retinal size arises from a shortened cell cycle time in the CMZ, and that Igf1r activation specifically increases proliferation of CMZ progenitors, but not CMZ retinal stem cells.

Overall, this is an interesting and well-written paper presenting convincing data and beautiful figures. This work provides evidence supporting a role for Igf signalling in the regulation of retinal tissue size and importantly, identifies a new marker to label and manipulate CMZ retinal stem cells specifically, allowing to study this cell population independently from progenitors in the same region, which has long been a challenge in the field. This will no doubt be of interest for many investigators. Conceptually, however, this paper is not unprecedented as Igf signalling has long been recognized as a regulator of organ/tissue size first in flies and more recently in vertebrates. Nonetheless, this is the first demonstration of a role for Igf signalling in the regulation of retinal growth and the work is well done, so I am overall positive about this paper. I have, however, some reservations on key aspects that I think need to be improved.

Comments for the author

1) The loss of function data is critical to formally demonstrate that Igf1r signalling is normally involved in retinal size control, which I think is an important point, but the evidence provided here is thin as it is entirely based on the use of one inhibitor of Igf1r (Fig. 1). While it may be a widely-used inhibitor, complementary genetic data is necessary to buttress the conclusion. It might also help the authors provide more definitive results that Igf1r signalling is actually required in the CMZ, because the current data do not allow to make this conclusion (the entire fish is incubated in the inhibitor), raising the possibility of non cell-autonomous effects.

2) Related to the point above, while the authors show reduced proliferation in the CMZ following incubation in the Igf1r inhibitor, they did not investigate whether this translates into smaller retinas. The authors report a 30% decrease in proliferation in the CMZ, which one would expect should translate into smaller retinas. Is this the case?

3) The in situ hybridization data presented in Figure S1 is not entirely convincing. Most of the front part of the eye (e.g. cornea) appears positive. Is this real labelling or some kind of edge effect, as is often seen with ISH?
It is important to exclude this possibility because the CMZ is located at the edge of the tissue and the staining might arise from the background “edge effect”.

4) Is it possible to provide some quantification of the % of progenitors in the CMZ that label for phospho-Igf1r (Fig. 1B)?

5) I found it a bit confusing that retinal region immediately adjacent to the CMZ is referred to as “central” retina. It is not really central, but more periphery.

6) The authors provide and “approximation of cell size” by quantifying the density of nuclei found in a 20 x 20µm square region and because they do not see changes in density, they conclude that Igf1r signalling does not affect cell size. I am not convinced this method is appropriate because it takes into account only the region around the nucleus of the cell, whereas many cells in the retina have processes that extend outside the INL and sometimes through the entire thickness of the retina (e.g. MG). In fact, given that the retina is thicker after activation of Igf1r signalling, it is very likely that Müller cells have a larger volume than in controls because they have to extend

© 2021. Published by The Company of Biologists under the terms of the Creative Commons Attribution License (https://creativecommons.org/licenses/by/4.0/).

processes over longer distances. The authors either need to provide a more accurate measure of cell volume, or leave out this data, as it is inconclusive in my opinion.

7) The authors classified all cells in the GCL as ganglion cells and they mention they deliberately neglected displaced amacrine cells (Fig. 3). It was not clear to me why they did this. Isn't it possible that the small increase in the number of cells in the GCL is due to displaced amacrines? It is also misleading to call these cells "RGCs", as these cells actually contain both RGCs and amacrines. RGC-specific markers could be used to distinguish RGCs from amacrines in the GCL.

8) The authors propose a model in which Igf1r signalling promotes proliferation of CMZ progenitors to increase retina size. But if this were the case, one would expect to find a proportional increase of all cell types. Yet, an interesting observation in this paper is that bipolar cells and amacrine cells are primarily overproduced. To me, this finding suggests that activating Igf1r signalling does more than simply promoting proliferation of the CMZ progenitors and might additionally favour specific cell fate decisions. This is not discussed in the paper. I think the author should consider adding a discussion of this point.

9) Although the ArCoS assay used in Fig. 5 was previously published by this group, I think it would be helpful to add a control showing what progenitor-derived clones look like. In the current figure, only stem cell-derived clone is shown, so it may be difficult for the reader to know exactly what to look for in the figure.

10) The sentence on lines 353-355 should be reformulated as it is unclear.

Reviewer 2

Advance summary and potential significance to field

The present paper addresses organ growth control, with a focus on the retina and Igf1r signaling. The authors found that constitutive activation of Igf1r signaling in the neurogenic zone of the medaka retina, the CMZ, leads to oversized retinas. Interestingly, however, retinal cell type composition of such big retinas is shifted toward interneurons. Moreover, the authors found that activation of Igf1r signaling in the CMZ preferentially expands the pool of progenitor cells but does not enlarge the stem cell population. The proposed mechanism to explain the phenotype involves changes in cell cycle length of retinal progenitors.

This work nicely contributes to our knowledge of the molecular mechanisms underlying stem and progenitor cell proliferation in the retina. The main claims of the manuscript are well supported by the data. The manuscript is very clear and nicely written. The illustrations are of very high quality and the experimental design very elegant. For instance, the authors employed a sophisticated lineage analysis tool that allowed them to discover a bona fide marker of CMZ stem cells that proved very useful for the present study.

Comments for the author

A few aspects require additional clarifications, however:

1. The authors highlighted many times in the manuscript that retinal growth can be uncoupled from overall body growth. It seems, however, quite expected that targeting the activation of a mitogenic signaling only in the retina would specifically affect the retina. Concluding about the role of Igf signaling in coordinating retina growth with body growth thus appears overstated. The title of the manuscript may thus need to be modified.

2. Igf1r-mediated signaling seems to be active only in single CMZ cells. Since the authors have identified Cndp as a bona fide marker for CMZ stem cells, it would be relevant to perform a co-labeling to identify whether cells in which Igf1r-mediated signaling is active are progenitor cells and not stem cells, in support with the proposed model.

© 2021. Published by The Company of Biologists under the terms of the Creative Commons Attribution License (https://creativecommons.org/licenses/by/4.0/).

3. The authors observed an increased number of PCNA+ cells in the CMZ of rx2::caigf1r retina compared to WT. They found that the cell cycle length of rx2::caigf1r progenitors was shorter. If such progenitors undergo the same number of cell divisions than in a control retina, they would generate new neurons more frequently but the number of PCNA+ would not be increased. Instead, one possibility to explain the increased number of progenitors is that they make additional rounds of cell cycles compared to wild type, like an amplification phase. Could the authors comment on this and, if appropriate, clarify their proposed model accordingly?

4. The authors provide quantifications with the number of BrdU cells in the CMZ. It would be useful to also provide the ratio of BrdU cells among PCNA+ cells. If G phases are shorter in rx2::caigf1r retina compared to controls, it is expected that BrdU/PCNA ratio would become higher.

5. A last issue is whether the oversized retina following activation of Igf1r signaling leads to changes in the visual acuity of the fish. This could be discussed as a perspective and be added to the very interesting discussion about the manuscript evolutionary implications.

Minor:
1. The altered cell type composition in oversized retinas could be mentioned in the abstract.

2. Line 417: Fig. 7I should be Fig. 6I

3. In Fig. S8C there is no control image.

Reviewer 3

Advance summary and potential significance to field

Becker et al. study the effect of modulating Igf1r downstream signalling in eye growth in medaka fish. They show that Igf1r components are expressed in the CMZ in medaka and that pIgf1r positive cells are located in the CMZ and MG cells of the retina. Furthermore, Igf1r inhibitor using an antagonist drug leads to less BrdU+ cells in the CMZ. They then go on and develop a transgenic line that expresses a constitutively active igf1r specifically in the CMZ of medaka, rx2::caigfl1r. The transgenic was validated by showing enhanced pAkt, a downstream effector of Igf1r.
The eyes in rx2::caigfl1r larvae and adult fish are bigger compared to wildtype embryos. Interestingly, the eye organisation in rx2::caigfl1r embryos remains comparable to a wt retina. The authors go on and show data that suggests that the size of the retina in rx2::caigfl1r transgenic is explained by an increase in the number of cells contributed by the CMZ and not from increased proliferation in the central retina not decreased apoptosis. Interestingly the proportion of amacrine and bipolar cells in the inner nuclear layer is significantly increased in rx2::caigfl1r retinas compared to wt conditions. The authors suggest that cell cycle is shorter in rx2::caigfl1r retinas, with S phase remaining the same and a double of BrdU+ cells observed in the CMZ, which could explain the bigger retinae in rx2::caigfl1r retinas.
To specifically enhance Igf1r activity in the stem cell niche of the CMZ the authors generated set of cndp transgenic lines, which drive expression in the cells in the CMZ likely to represent the retina stem cells. By using the GaudiRDG system previously developed in their lab, the authors confirm that the cndp driver is expressing the transgenes in the CMZ stem cells as the expected ArCoS are formed and not clonal footprints which would be the consequence of CMZ progenitor cell expression. The authors go on and show that the effect of Igf1r activation over the retina size is exclusively mediated by CMZ progenitor cells and not stem cells as cndp::caigfl1r retinas are not bigger than wt ones.

Comments for the author

Overall, I think this manuscript presents a set of interesting results regarding the function of Igf1r mediated signalling activity in the growing retina of medaka fish. The data suggests that this function could explain a coupling of the size of the eye and the body during the life of this fish. However, I think it is a little too far reached to suggest that they have studied how organ growth is coordinated with body growth in this species as they repeatedly insist throughout the manuscript. For example, is there an accurate and deep description showing that the eye and body size ratio is

© 2021. Published by The Company of Biologists under the terms of the Creative Commons Attribution License (https://creativecommons.org/licenses/by/4.0/).

constant or maintained during the life of medaka such that we can argue assume such coupling or coordination exists? I do think that this manuscript would benefit from restricting the interpretation of the results from a grand broad biological statement to a more accurate and conceptually restricted description of the phenomenon they observe.

Major suggestions:

1. The authors show that it is single cells in the CMZ progenitor domain that are detected using anti phosphorylated Igf1r antibodies. Can such few cells mediating Igf1r function in the CMZ explain the effect observed when inhibiting or activating this pathway? This should be taken into account for the interpretation of the results and I would expect the authors to comment in this particular observation. Are these pIgf1r+ cells also pAkt+ like when using the rx2::caigf1r transgenic? I think it is important to provide more data regarding this issue to fully support the argument that it is igf1r downstream activity that is mediating the effect observed in the rx2::caigfl1r transgenic. Are there any other Igf1r downstream readouts that could be evaluated?

2. Rx2::caigfl1r transgenic eyes are bigger in larvae but the manuscript does not show any quantitative data and only mentions a "prominent oversized retina" (line 158) and that the "relative eye size was significantly increased (line 160).
Rather than adjectives, can the authors please present the objective quantified information, for example, the fold change of the rx2::caigfl1r relative to the wt condition. I also think the authors should include tables with the raw data.
Furthermore, what kind of statistical test was applied to determine significance in these experiments? A few statistical tests are mentioned in materials and methods, but it is not clear which of those were used to analyse eye size change or other datasets.

3. The rx2::caigfl1r retina in Figure 2G seems a lot bigger than the wt one in Fig 2F, which makes the point for the argument, but is not a good reflection of the data shown in the plot Fig 2D. Can the authors please point out if the retinas in Fig 2F and G are outliers or are representative of one of the samples in the median? I would suggest marking which of the samples in the raw data corresponds to the images in the panels in Fig 2F and G. Reinforcing my observation, the rx2::caigfl1r retinas in Fig S4 look a lot more consistent with the data in plot Fig 2D.

4. The authors argue that the transgenic cndp reporter lines recapitulate the mRNA expression (Line 324). However, they show no in situ hybridisation data for cndp which is necessary to back up such argument. Can the authors please include this data in the manuscript?

Minor suggestions:

1. The sentence in line 40 "How retinal growth is regulated and can be uncoupled from body growth in different fish species to achieve differential eye sizes and function is not understood" has a strange logic to it. Why is it assumed that there is a need for retina growth to be uncoupled from body growth? Not clear to me.

2. The authors mention "homeostasis of optical parameters" in line 49. Can there be such homeostasis of optical parameters? Which optical parameters are the authors referring to?

3. In line 75, it seems strange to me to refer to the localisation of Igf1 "receptor binding sites" rather than just the 'Igf1 receptor localisation'.

4. In line 82 the authors state that they: "…address the coordination of retinal size with body growth". It is not clear to me that this is the case with the set of experiments in this manuscript and suggest the statement should be toned down.

5. Line 86, is it the increase in the size of the eye really 'prominent'? I suggest avoiding such adjectives and let the reader make their mind about the effect the authors observe. Especially considering that the authors do not mention the quantification of this size difference.

6. Line 90, the authors mention a "tightly coordinated program", but they do not state the nature of such a program. Differentiation programme? Does this result really say something about a tightly

© 2021. Published by The Company of Biologists under the terms of the Creative Commons Attribution License (https://creativecommons.org/licenses/by/4.0/).

coordinated differentiation programme? I'm not so sure. Could the authors please explain this statement?

7. Fig1 Panels C and D, the difference in BrdU labelling between the experimental and control is not very evident. Maybe include the image that only shows the fluorescent panel line in Fig1 B?

8. The Igf1r antagonist drug the authors use is not specific to Igf1r and can also inhibit Insulin receptor activity which is also shown to be expressed in the CMZ.
Can the authors please comment on this and also tone the arguments considering this caveat.

9. Line 118, I would suggest including a citation for the Igf1r activation mechanism.

10. Line 119, I am not sure it is right to say you "stained the phosphorylated Igf1r (pIgf1r) by immunohistochemistry" …. maybe better say that you "detected the phosphorylated Igf1r"

11. In Line 126 the authors mention that BrdU+ cells are cells in S-phase.
However, the BrdU incubation time used in these and other experiments would suggest that it is cells that are in or have gone through S phase are BrdU positive. Can the authors please check this?

12. The use of the expression "retina topology" in line 189 can be ambiguous maybe use another more accurate description.

13. As mentioned above, I think it is better to avoid adjectives like 'enormous' (line 211) when describing the data and stick to objective descriptions and let the reader make their mind regarding the interpretation of the data.

14. The authors state that experiments were performed at hatchling larvae or adult fish. Can they please state the stages in hours, days or months post fertilisation for accuracy of the descriptions?

First revision

Author response to reviewers' comments

Reviewer 1 Advance Summary and Potential Significance to Field:
In this paper, Becker et al. investigate the role of Igf signalling in the regulation of tissue size, more specifically in the medaka fish retina. They find that inhibition of Igf1r- mediated signalling using a small molecule inhibitor leads to reduced proliferation in the CMZ. Conversely, targeted activation of Igf1r signalling in the CMZ increases proliferation, which leads to increased retina and eye size, without affecting the overall size of the fish. Interestingly, the larger retinas observed following activation of Igf1r in the CMZ contain more retinal ganglion cells, amacrine cells, and bipolar cells, but no change in other cell types. The authors further show that the increased retinal size arises from a shortened cell cycle time in the CMZ, and that Igf1r activation specifically increases proliferation of CMZ progenitors, but not CMZ retinal stem cells.

Overall, this is an interesting and well-written paper presenting convincing data and beautiful figures. This work provides evidence supporting a role for Igf signalling in the regulation of retinal tissue size and, importantly, identifies a new marker to label and manipulate CMZ retinal stem cells specifically, allowing to study this cell population independently from progenitors in the same region, which has long been a challenge in the field. This will no doubt be of interest for many investigators. Conceptually, however, this paper is not unprecedented as Igf signalling has long been recognized as a regulator of organ/tissue size, first in flies and more recently in vertebrates. Nonetheless, this is the first demonstration of a role for Igf signalling in the regulation of retinal growth and the work is well done, so I am overall positive about this paper. I have, however, some reservations on key aspects that I think need to be improved.

© 2021. Published by The Company of Biologists under the terms of the Creative Commons Attribution License (https://creativecommons.org/licenses/by/4.0/).

Reviewer 1 Comments for the Author:

1. The loss of function data is critical to formally demonstrate that Igf1r signalling is normally involved in retinal size control, which I think is an important point, but the evidence provided here is thin as it is entirely based on the use of one inhibitor of Igf1r (Fig. 1). While it may be a widely-used inhibitor, complementary genetic data is necessary to buttress the conclusion. It might also help the authors provide more definitive results that Igf1r signalling is actually required in the CMZ, because the current data do not allow to make this conclusion (the entire fish is incubated in the inhibitor), raising the possibility of non cell-autonomous effects.
The reviewer raises an interesting point that we have tried addressing by extending the range of treatments with the well-established inhibitor. While we agree with the reviewer that for addressing this specific aspect, the most conclusive experiment would be a conditional knockout of Igf1r in the retina, even in the days of Crispr/Cas9 a retina specific conditional inactivation is experimentally challenging. Such an approach has not been established in medaka and is unfortunately not feasible within the time available for the revision.
We explored the specific expression of dominant negative receptor variants as an alternative, but the leakiness and mosaicism of the drivers in the injected generation unfortunately mandates the establishment of stable lines which again takes at least three to four months. Our major concern with such an experiment is the lack of a CMZ progenitor-specific driver. Á dominant negative construct expressed under control of the rx2 promoter will likely have a strong negative impact on retinal development. Due to rx2 expression from the optic vesicle stage onward we would expect that generation of the postembryonic retina and establishment of the CMZ would be severely affected, thereby further hindering an informative experimental outcome.
As an alternative, we have therefore extended and modified the inhibitor treatment aiming at further exploiting this assay. However, under the given conditions, even control treated hatchlings do neither grow in length nor in eye diameter (see table below). We have amended the manuscript accordingly, toned down our conclusion and take care to discuss the limitations and caveats of this experiment. We state in the paragraph following the inhibitor data that we used this experiment as starting point for further studies and not as an equivalent to a conditional loss of function experiment.

average	***eye diameter [mm]***	***body length [mm]***	***ratio eye diameter/body length***
2d dmso	*0.404*	*5.113*	*0.079*
3d dmso	*0.402*	*5.114*	*0.079*
4d dmso	*0.413*	*5.165*	*0.080*
5d dmso	*0.406*	*5.117*	*0.079*
6d dmso	*0.407*	*5.147*	*0.079*
2d inhibitor	*0.405*	*5.064*	*0.080*
3d inhibitor	*0.406*	*5.056*	*0.080*
4d inhibitor	*0.405*	*5.025*	*0.081*
5d inhibitor	*0.400*	*4.974*	*0.080*
6d inhibitor	*0.398*	*4.925*	*0.081 (excluding 2 dead fish)*

2. Related to the point above, while the authors show reduced proliferation in the CMZ following incubation in the Igf1r inhibitor, they did not investigate whether this translates into smaller retinas. The authors report a 30% decrease in proliferation in the CMZ, which one would expect should translate into smaller retinas. Is this the case?
We thank the referee for this interesting question that we had not explicitly addressed in the submitted version. We have addressed it in two ways and have included part of it in the revised version of the manuscript:
On the one hand we have quantified the relative eye size of inhibitor- and control- treated hatchlings and have included these quantifications in the revised manuscript (Fig. S2A). Neither eye size nor body length of inhibitor-treated fish was decreased compared to control fish, which is likely due to the fact that the treatment was only administered for 24 hours.
On the other hand, we have treated embryos for a longer time period to exceed the average doubling time of retinal progenitor cells and have tried to extend it for the time an early progenitor

© 2021. Published by The Company of Biologists under the terms of the Creative Commons Attribution License (https://creativecommons.org/licenses/by/4.0/).

takes to terminal differentiation (5-7 days). As outlined in the previous point, under the given conditions, even control treated hatchlings do neither grow in length nor in eye diameter and inhibitor-treated fish died after 6- 7 days. During the first weeks after hatch, space, oxygen and food availability are all critical factors for rapid growth, which cannot be reasonably met in this assay and would require using a large amount of inhibitor.

3. The in situ hybridization data presented in Figure S1 is not entirely convincing. Most of the front part of the eye (e.g. cornea) appears positive. Is this real labelling or some kind of edge effect, as is often seen with ISH? It is important to exclude this possibility because the CMZ is located at the edge of the tissue and the staining might arise from the background "edge effect".
We thank the reviewer for this comment. While we are aware of the possibility that background at the edge can arise, we are convinced that the ISH signal in the CMZ in the images in Figure S1, which were taken on sections of the retina to limit potential edge effects, is a reflection of the actual expression. We have repeated the analysis and provide a new panel in Figure S1. To further strengthen this point we have attached the ISH for the downstream target foxo3. Foxo3 is expressed in late progenitor cells as well as cells in the differentiated retina but does not show any staining in the stem cell region of the CMZ.

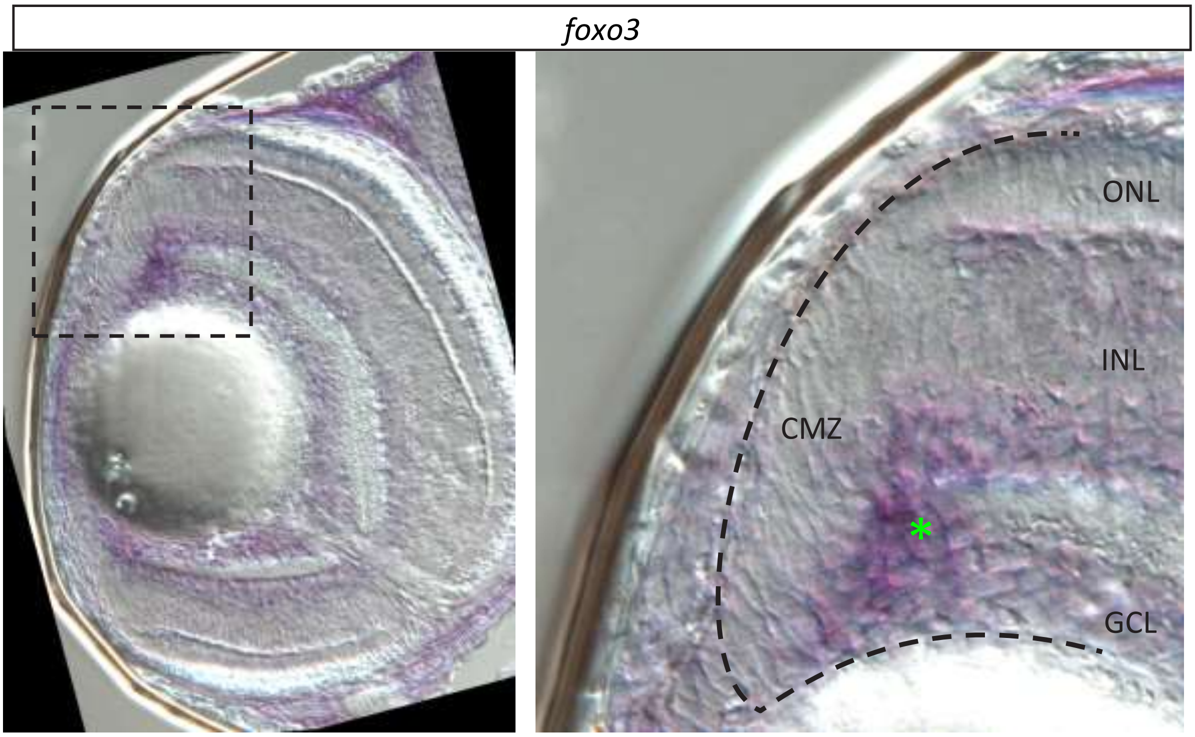

4. Is it possible to provide some quantification of the % of progenitors in the CMZ that label for phospho-Igf1r (Fig. 1B)?
We are providing the requested information in the revised version of the manuscript. Reviewer #2 had requested the quantification of pIgf1r-positive cells in the context of the cndp reporter line to show that these cells are indeed progenitor cells. We now provide more detailed quantifications of pIgf1r-positive cells in Fig S6E,F of the revised version of the manuscript.
In addition, and to further clarify this important point, we have estimated an average number of progenitor cells per CMZ section (from our Pcna data in the cell cycle experiment) and provide the percentage of pIgf1r cells relative to this number. This indicates the fraction of pIgf1r-positive cells at the sampling timepoint and is represented as "(around 2%, estimation based on quantification of Pcna-positive cells in Fig. 4 and Fig. S5)" in the revised manuscript.

5. I found it a bit confusing that retinal region immediately adjacent to the CMZ is referred to as "central" retina. It is not really central, but more periphery.
The reviewer is right and we have taken care to precisely describe the respective domains in the

© 2021. Published by The Company of Biologists under the terms of the Creative Commons Attribution License (https://creativecommons.org/licenses/by/4.0/).

revised manuscript. We have changed the description in the text to "(peripheral) differentiated/laminated retina" and only used "central" as description where either the entire differentiated retina or the central-most retina region is indicated to avoid any confusion.

6. The authors provide and "approximation of cell size" by quantifying the density of nuclei found in a 20 x 20µm square region and because they do not see changes in density, they conclude that Igf1r signalling does not affect cell size. I am not convinced this method is appropriate because it takes into account only the region around the nucleus of the cell, whereas many cells in the retina have processes that extend outside the INL and sometimes through the entire thickness of the retina (e.g. MG). In fact, given that the retina is thicker after activation of Igf1r signalling, it is very likely that Müller cells have a larger volume than in controls because they have to extend processes over longer distances. The authors either need to provide a more accurate measure of cell volume, or leave out this data, as it is inconclusive in my opinion.
We see the point raised by the reviewer and followed their advice to leave out these potentially confusing data. We accordingly deleted it from the revised manuscript.

7. The authors classified all cells in the GCL as ganglion cells and they mention they deliberately neglected displaced amacrine cells (Fig. 3). It was not clear to me why they did this. Isn't it possible that the small increase in the number of cells in the GCL is due to displaced amacrines? It is also misleading to call these cells "RGCs", as these cells actually contain both RGCs and amacrines. RGC-specific markers could be used to distinguish RGCs from amacrines in the GCL.
We see the point of the referee and apparently our motivation for this grouping in the submitted version has not been apparent. We cannot discriminate between RGCs and displaced amacrine cells due to the lack of suitable markers in medaka. Up to date, we have not identified expression reporters or antibodies in medaka that allow us to clearly distinguish these cell types. To clarify this and to avoid misleading the reader, we now describe the "cells in the GCL" summing up RGCs and displaced amacrine cells. We have revised text, figure legends and figures accordingly.

8. The authors propose a model in which Igf1r signalling promotes proliferation of CMZ progenitors to increase retina size. But if this were the case, one would expect to find a proportional increase of all cell types. Yet, an interesting observation in this paper is that bipolar cells and amacrine cells are primarily overproduced. To me, this finding suggests that activating Igf1r signalling does more than simply promoting proliferation of the CMZ progenitors and might additionally favour specific cell fate decisions. This is not discussed in the paper. I think the author should consider adding a discussion of this point.
We thank the reviewer for pointing this out. Following the suggestion we have added a paragraph to the discussion placing those results in context: "In the teleost retina, several populations of lineage-specified progenitors reside in the CMZ, and modification of their transcriptional signatures shifts cell type ratios (Pérez Saturnino et al., 2018). The activation of Igf1r signaling elicits a pronounced increase in ACs and BCs, suggesting that Igf1r activation might either convey certain cell fates or favor expansion of a specific progenitor population lineage- committed to generate INL cells."

9. Although the ArCoS assay used in Fig. 5 was previously published by this group, I think it would be helpful to add a control showing what progenitor-derived clones look like. In the current figure, only stem cell-derived clone is shown, so it may be difficult for the reader to know exactly what to look for in the figure.
We have been following the reviewer's advice and have added a scheme depicting stem cell- and progenitor-derived clones to the figure (Fig. 5D) to allow the reader to instantly appreciate and interpret the results of the lineage tracing analysis.

10. The sentence on lines 353-355 should be reformulated as it is unclear.
We have adjusted the sentence in the revised manuscript to clarify our rationale for this experiment: "To address whether stem or progenitor cell populations are expanded in response to activated Igf1r signaling we took advantage of the stem cell-specific expression of cndp and used rx2 as marker for stem and progenitor cells (Fig. 6A)."

Reviewer 2 Advance Summary and Potential Significance to Field:
The present paper addresses organ growth control, with a focus on the retina and Igf1r signaling. The authors found that constitutive activation of Igf1r signaling in the neurogenic zone of the medaka

© 2021. Published by The Company of Biologists under the terms of the Creative Commons Attribution License (https://creativecommons.org/licenses/by/4.0/).

retina, the CMZ, leads to oversized retinas. Interestingly, however, retinal cell type composition of such big retinas is shifted toward interneurons. Moreover, the authors found that activation of Igf1r signaling in the CMZ preferentially expands the pool of progenitor cells but does not enlarge the stem cell population. The proposed mechanism to explain the phenotype involves changes in cell cycle length of retinal progenitors.

This work nicely contributes to our knowledge of the molecular mechanisms underlying stem and progenitor cell proliferation in the retina. The main claims of the manuscript are well supported by the data. The manuscript is very clear and nicely written. The illustrations are of very high quality and the experimental design very elegant. For instance, the authors employed a sophisticated lineage analysis tool that allowed them to discover a bona fide marker of CMZ stem cells that proved very useful for the present study.

Reviewer 2 Comments for the Author:
A few aspects require additional clarifications, however:

1. The authors highlighted many times in the manuscript that retinal growth can be uncoupled from overall body growth. It seems, however, quite expected that targeting the activation of a mitogenic signaling only in the retina would specifically affect the retina. Concluding about the role of Igf signaling in coordinating retina growth with body growth thus appears overstated. The title of the manuscript may thus need to be modified.
Under normal growth conditions, the retina size and total body size are tightly coordinated. A general mitogenic trigger as for example provided by the expression of an activated oncogene kras^{12V} in the same manner (driven by the same rx2 driver element and vector backbone) did not result in any phenotype. We had not included these negative results in the initially submitted manuscript. However, in light of the point raised by the referee we are ready to incorporate these negative results as supplementary data into the revised version of the manuscript to strengthen the specificity of the Igf signaling effect if requested.
We feel that the title is a fair reflection of our findings given the specific expression and specific effect of the Igf signaling cascade in the proliferative domain of the eye.

2. Igf1r-mediated signaling seems to be active only in single CMZ cells. Since the authors have identified Cndp as a bona fide marker for CMZ stem cells, it would be relevant to perform a co-labeling to identify whether cells in which Igf1r-mediated signaling is active are progenitor cells and not stem cells, in support with the proposed model.
We have been following the reviewer's advice and have performed a co-label experiment of pIgf1r in the cndp::eGFP-caax reporter line to address if any of the stem cells show signs of active Igf signaling. We did not observe any co-labeling and have added the new panels E,F to Fig. S6 and this sentence to the main text: "In line with this observation, pIgf1r staining indicative of the activation of the Igf signaling cascade was only detected in progenitor cells, but not in cndp-positive stem cells (Fig. S6E,F)."

3. The authors observed an increased number of PCNA+ cells in the CMZ of rx2::caigf1r retina compared to WT. They found that the cell cycle length of rx2::caigf1r progenitors was shorter. If such progenitors undergo the same number of cell divisions than in a control retina, they would generate new neurons more frequently but the number of PCNA+ would not be increased. Instead, one possibility to explain the increased number of progenitors is that they make additional rounds of cell cycles compared to wild type, like an amplification phase. Could the authors comment on this and, if appropriate, clarify their proposed model accordingly?
The reviewer raises an interesting point concerning the division modes of retinal progenitor cells in the CMZ. Indeed, retinal progenitors were shown to undergo a mixture of symmetric proliferative, asymmetric and symmetric differentiative divisions, likely depending on the stage of the progenitor cells (Wan et al., 2016). The fact that we observe an increase in Pcna-positive cells along with shortening of the cell cycle indicates that self-renewing divisions of the progenitor cells are increased. This can also be supported by the fact that the rx2-positive cell population, but not the cndp-positive population is enlarged. We have included a paragraph in the discussion addressing this point.

4. The authors provide quantifications with the number of BrdU cells in the CMZ. It would be useful to also provide the ratio of BrdU cells among PCNA+ cells. If G phases are shorter in rx2::caigf1r retina

© 2021. Published by The Company of Biologists under the terms of the Creative Commons Attribution License (https://creativecommons.org/licenses/by/4.0/).

compared to controls, it is expected that BrdU/PCNA ratio would become higher.
We thank the reviewer for this suggestion and have added the following sentence including the ratios to the cell cycle analysis paragraph: "The ratio of Brdu- to Pcna- positive cells increased from 50% in wildtype to 57% in rx2::caigf1r hatchlings in the dorsal CMZ, and from 55% to 60% in the ventral CMZ, indicating shortened G phases in the rx2::caigf1r CMZ."

5. A last issue is whether the oversized retina following activation of Igf1r signaling leads to changes in the visual acuity of the fish. This could be discussed as a perspective and be added to the very interesting discussion about the manuscript evolutionary implications.
We are grateful for bringing this up and have followed the reviewer's advice and included a corresponding paragraph in the discussion of the revised version of the manuscript.

Minor:

1. The altered cell type composition in oversized retinas could be mentioned in the abstract.
We have alluded to this in the last sentence of the abstract "Our findings position Igf signaling as key module for controlling retinal size and composition with important evolutionary implications."

2. Line 417: Fig. 7I should be Fig. 6I
We have corrected that oversight.

3. In Fig. S8C there is no control image.
We have edited Fig. S8 (now Fig. S7) to include a control image (Fig. S7C,D).

<u>Reviewer 3</u> Advance Summary and Potential Significance to Field:
Becker et al. study the effect of modulating Igf1r downstream signalling in eye growth in medaka fish. They show that Igf1r components are expressed in the CMZ in medaka and that pIgf1r positive cells are located in the CMZ and MG cells of the retina. Furthermore, Igf1r inhibitor using an antagonist drug leads to less BrdU+ cells in the CMZ. They then go on and develop a transgenic line that expresses a constitutively active igf1r specifically in the CMZ of medaka, rx2::caigfl1r. The transgenic was validated by showing enhanced pAkt, a downstream effector of Igf1r. The eyes in rx2::caigfl1r larvae and adult fish are bigger compared to wildtype embryos. Interestingly, the eye organisation in rx2::caigfl1r embryos remains comparable to a wt retina.
The authors go on and show data that suggests that the size of the retina in rx2::caigfl1r transgenic is explained by an increase in the number of cells contributed by the CMZ and not from increased proliferation in the central retina not decreased apoptosis. Interestingly the proportion of amacrine and bipolar cells in the inner nuclear layer is significantly increased in rx2::caigfl1r retinas compared to wt conditions. The authors suggest that cell cycle is shorter in rx2::caigfl1r retinas, with S phase remaining the same and a double of BrdU+ cells observed in the CMZ, which could explain the bigger retinae in rx2::caigfl1r retinas.
To specifically enhance Igf1r activity in the stem cell niche of the CMZ the authors generated set of cndp transgenic lines, which drive expression in the cells in theCMZ likely to represent the retina stem cells. By using the GaudiRDG system previously developed in their lab, the authors confirm that the cndp driver is expressing the transgenes in the CMZ stem cells as the expected ArCoS are formed and not clonal footprints which would be the consequence of CMZ progenitor cell expression. The authors go on and show that the effect of Igf1r activation over the retina size is exclusively mediated by CMZ progenitor cells and not stem cells as cndp::caigfl1r retinas are not bigger than wt ones.

Reviewer 3 Comments for the Author:
Overall, I think this manuscript presents a set of interesting results regarding the function of Igf1r mediated signalling activity in the growing retina of medaka fish. The data suggests that this function could explain a coupling of the size of the eye and the body during the life of this fish. However, I think it is a little too far reached to suggest that they have studied how organ growth is coordinated with body growth in this species as they repeatedly insist throughout the manuscript. For example, is there an accurate and deep description showing that the eye and body size ratio is constantor maintained during the life of medaka, such that we can argue assume such coupling or coordination exists? I do think that this manuscript would benefit from restricting the interpretation of the results from a grand broad biological statement to a more accurate and conceptually restricted description of the phenomenon they observe.

© 2021. Published by The Company of Biologists under the terms of the Creative Commons Attribution License (https://creativecommons.org/licenses/by/4.0/).

Major suggestions:

1. The authors show that it is single cells in the CMZ progenitor domain that are detected using anti phosphorylated Igf1r antibodies. Can such few cells mediating Igf1r function in the CMZ explain the effect observed when inhibiting or activating this pathway? This should be taken into account for the interpretation of the results and I would expect the authors to comment in this particular observation. Are these pIgf1r+ cells also pAkt+ like when using the rx2::caigfl1r transgenic? I think it is important to provide more data regarding this issue to fully support the argument that it is igf1r downstream activity that is mediating the effect observed in the rx2::caigfl1r transgenic. Are there any other Igf1r downstream readouts that could be evaluated?
The referee raises an important point that we had been carefully addressing in the submitted version of the manuscript. Previous studies using the caIgf1r fusion receptor validated signal transduction by assaying pAkt levels, which we did as well for our specific transgenic line. We have added this information to the text: "The ability of the caIgf1r fusion receptor to induce signaling transduction via the PI3K/Akt axis has previously been validated in several studies by assaying pAkt levels (Carboni et al., 2005; Gusscott et al., 2016; Pappano et al., 2009)."
We also have attempted to co-label the activated Igf1r together with pAkt as indicator for pathway activation, but unfortunately the only antibodies reliably working in medaka are both raised in rabbit, which makes it impossible to address this point.
In light of published data and given that assaying phosphorylation of Akt (pAkt levels) represents the routinely used approach for evaluating Igf1r downstream signaling activity, we are convinced that the co-localization of caigf1r expression and the pAkt signal, together with the distinct increase in the pAkt signal in those retinae, represent a clear argument for the constitutive active receptor triggering pathway activity also in medaka.
Concerning the sparsity of the pIgf1r-positive cells we have revised the text to clearly emphasize that this is just a snapshot of cells which at the moment of fixation were actively signaling via Igf1r, thus being pIgf1r-positive. We have added the following sentence: "The sparse distribution of pIgf1r-positive cells likely reflects a snapshot of the highly dynamic process of the activation and phosphorylation of receptor tyrosine kinases (Kiyatkin et al., 2020; Varkaris et al., 2013)."

2. Rx2::caigfl1r transgenic eyes are bigger in larvae but the manuscript does not show any quantitative data and only mentions a "prominent oversized retina" (line 158) and that the "relative eye size was significantly increased (line 160). Rather than adjectives, can the authors please present the objective quantified information, for example, the fold change of the rx2::caigfl1r relative to the wt condition. I also think the authors should include tables with the raw data. Furthermore, what kind of statistical test was applied to determine significance in these experiments? A few statistical tests are mentioned in materials and methods, but it is not clear which of those were used to analyse eye size change or other datasets.
We thank the reviewer for this comment. We quantify the relative eye size in Fig. 2D and E for hatchlings and adults respectively, and we have revised the text according to the suggestions (see also answers to minor suggestions 5 & 13). As suggested, we have included the raw data on eye size quantifications (Tables S1,2) into the supplement.
Additionally, we have specified which statistical test was used for analysis in the figure legend of each plot.

3. The rx2::caigfl1r retina in Figure 2G seems a lot bigger than the wt one in Fig 2F, which makes the point for the argument, but is not a good reflection of the data shown in the plot Fig 2D. Can the authors please point out if the retinas in Fig 2F and G are outliers or are representative of one of the samples in the median? I would suggest marking which of the samples in the raw data corresponds to the images in the panels in Fig 2F and G. Reinforcing my observation, the rx2::caigfl1r retinas in Fig S4 look a lot more consistent with the data in plot Fig 2D.
We have analyzed the height of the retinal columns in dorsal, ventral and central positions of the retinae in Fig. 2F,G and compared them with the medians from Fig. 2H, and values from both wildtype and rx2::caigf1r are representative of or very close to the median values.

© 2021. Published by The Company of Biologists under the terms of the Creative Commons Attribution License (https://creativecommons.org/licenses/by/4.0/).

retinal thickness	dorsal [µm]	ventral [µm]	central [µm]
**wt Fig. 2F**	88.56	78.98	75.06
**wt median**	87.48	80.10	75.15
***rx2::caigf1r* Fig. 2G**	107.43	98.86	85.59
***rx2::caigf1r* median**	106.82	98.51	84.52

4. The authors argue that the transgenic cndp reporter lines recapitulate the mRNA expression (Line 324). However, they show no in situ hybridisation data for cndp, which is necessary to back up such argument. Can the authors please include this data in the manuscript?
We have edited Figure S6 to include the new panels B,C, where we show a whole- mount in situ hybridization for cndp on a stage 32 medaka embryo and an antibody staining of a stage 32 cndp::eGFP-caax reporter embryo to underline the recapitulation of cndp expression in the reporter.

Minor suggestions:

1. The sentence in line 40 "How retinal growth is regulated and can be uncoupled from body growth in different fish species to achieve differential eye sizes and function is not understood" has a strange logic to it. Why is it assumed that there is a need for retina growth to be uncoupled from body growth? Not clear to me.
Different fish species display different relative eye sizes. This statement is not meant to say that within one fish, there will be an uncoupling of retina and body growth, but rather from an evolutionary point of view – looking at the whole teleost clade – that how differences in size of eyes or other organs arise is not clear yet. We have changed this sentence accordingly: "How retinal growth is regulated in different fish species to achieve differential eye sizes and function is not understood."

2. The authors mention "homeostasis of optical parameters" in line 49. Can there be such homeostasis of optical parameters? Which optical parameters are the authors referring to?
Important optical parameters are for example the density of photoreceptors, the matching of the focal point (depending on lens size and diameter) onto the photoreceptor segments, and photoreceptor to ganglion cell convergence. Throughout postembryonic growth, it is important that these parameters are aligned with each other to ensure functionality. This does not necessarily mean that these parameters won't change throughout life, but rather that any changes to one parameter will possibly result in changes to the other ones as well in order for the organ to continue being a functional unit. We have included this clarification into the sentence of the introduction.

3. In line 75, it seems strange to me to refer to the localisation of Igf1 "receptor binding sites" rather than just the 'Igf1 receptor localisation'.
We have changed the sentence accordingly: "its receptor is located in the CMZ".

4. In line 82 the authors state that they: "…address the coordination of retinal size with body growth". It is not clear to me that this is the case with the set of experiments in this manuscript and suggest the statement should be toned down.
We do think that this statement is valid, based on the experiments presented here, as Igf1r signaling over-activation impacts on retinal proliferation and organ size, and Igf signaling inhibition decreases proliferation. Hence we show that retinal growth, which is normally coordinated with body growth, can be uncoupled and increased independent from the growth rate of the rest of the body.

5. Line 86, is it the increase in the size of the eye really 'prominent'? I suggest avoiding such adjectives and let the reader make their mind about the effect the authors observe. Especially considering that the authors do not mention the quantification of this size difference.
We have always evaluated size very conservatively using one dimension, the diameter, as a proxy.

© 2021. Published by The Company of Biologists under the terms of the Creative Commons Attribution License (https://creativecommons.org/licenses/by/4.0/).

Instead the eyes grow in three dimensions and indeed the increase in cell numbers is prominent. The referee is right that when just using one dimension, as we did, the term prominent is not appropriate. We have therefore deleted "prominent" in this sentence and revised the rest of the manuscript according when describing the size effect. We have now included quantification of the size difference in the results paragraph where we explain this experiment in the context of the transgenic line and in more detail.

6. Line 90, the authors mention a "tightly coordinated program", but they do not state the nature of such a program. Differentiation programme? Does this result really say something about a tightly coordinated differentiation programme? I'm not so sure. Could the authors please explain this statement?
We thank the reviewer for this comment and have toned down this statement accordingly.

7. Fig1 Panels C and D, the difference in BrdU labelling between the experimental and control is not very evident. Maybe include the image that only shows the fluorescent panel line in Fig1 B?
We agree that the difference between ~80 and ~60 labeled cells is not easy to see. We have changed the DAPI channel to blue, which enhances the visibility of the green BrdU staining while keeping all positional and structural information provided by the DAPI staining.

8. The Igf1r antagonist drug the authors use is not specific to Igf1r and can also inhibit Insulin receptor activity which is also shown to be expressed in the CMZ. Can the authors please comment on this and also tone the arguments considering this caveat.
We have added/adapted the following sentences in the inhibitor paragraph: "While the NVP-AEW541 inhibitor displays a high specificity for Igf1r, an impact on Insr and other related kinases cannot be ruled out (Attias-Geva et al., 2011; García- Echeverría et al., 2004).";
"…indicating that Igf/insulin signaling contributes to regulating proliferation in the CMZ.";
"These results suggest that ligands and receptors of the Igf/insulin signaling cascade expressed in the CMZ contribute to CMZ proliferation."

9. Line 118, I would suggest including a citation for the Igf1r activation mechanism.
We included a citation as suggested: (Laviola et al., 2007).

10. Line 119, I am not sure it is right to say you "stained the phosphorylated Igf1r (pIgf1r) by immunohistochemistry" …. maybe better say that you "detected the phosphorylated Igf1r"
We thank the reviewer for this suggestion and have changed the sentence accordingly.

11. In Line 126 the authors mention that BrdU+ cells are cells in S-phase. However, the BrdU incubation time used in these and other experiments would suggest that it is cells that are in or have gone through S phase are BrdU positive. Can the authors please check this?
The reviewer is absolutely right, and we changed the sentence accordingly: "…and BrdU to detect proliferatively active cells that entered or went through S phase during the incubation time."

12. The use of the expression "retina topology" in line 189 can be ambiguous, maybe use another more accurate description.
To avoid any ambiguity, we have replaced "topology" with "architecture".

13. As mentioned above, I think it is better to avoid adjectives like 'enormous' (line 211) when describing the data and stick to objective descriptions and let the reader make their mind regarding the interpretation of the data.
We have deleted adjectives such as "enormous", and added the percentage eye size increase at first mention of said increase.

14. The authors state that experiments were performed at hatchling larvae or adult fish. Can they please state the stages in hours, days or months post fertilisation for accuracy of the descriptions?
Staging with days or hours post fertilization is not used in medaka fish, since their development is dependent on temperature. The medaka community rather follows

a precise, temperature-independent staging table relying on the appearance of defined morphological features as published in (Iwamatsu, 2004) for staging. Hatchling stage reflects that these fish are newly hatched larvae or stage 40 according to (Iwamatsu, 2004), which we have now

© 2021. Published by The Company of Biologists under the terms of the Creative Commons Attribution License (https://creativecommons.org/licenses/by/4.0/).

added at first mention of hatchling stage in the text and in Material & Methods: "Fish at hatching stage (Iwamatsu stage 40 (Iwamatsu, 2004))".
We have also specified the age of adult fish by including "3-month-old" in the figure legends where adult fish are mentioned.

Second decision letter

MS ID#: DEVELOP/2020/199133

MS TITLE: Igf signaling coordinates retina growth with body growth by modulating progenitor cell division

AUTHORS: Clara Becker, Katharina Lust, and Joachim Wittbrodt

I have now received all the referees reports on the above manuscript, and have reached a decision. The referees' comments are appended below, or you can access them online: please go to BenchPress and click on the 'Manuscripts with Decisions' queue in the Author Area.

The overall evaluation is positive and there are just a few relatively minor comments to address before we proceed to publication.

Reviewer 1

Advance summary and potential significance to field

I was a little disappointed that he authors could not offer better data on the loss of function studies, as I think it would have strengthen the paper significantly, but I understand the technical limitations involved. I appreciate their efforts to expend the data with the inhibitor and to tone down their conclusions as an alternative. I think this is reasonable. My other points have been addressed appropriately by addition of new data or text changes. I have no more issues to flag in this paper. Congratulations to the authors for a nice study.

Comments for the author

None

Reviewer 2

Advance summary and potential significance to field

The authors have satisfactorily addressed my previous comments. The additional experiment shown in Figure S6E,F supports and strengthens their previous findings. The authors now also provide ratios of BrdU/PCNA in support of their cell cycle length data and have enriched their discussion regarding the division mode of retinal progenitors in the CMZ of rx2::caigf1r retina. Regarding my previous comment 1, although I believe that the question could still be debatable, I consider that it does not require any additional data. I therefore consider that the revised manuscript is perfectly suitable for publication in Development.

Comments for the author

no suggestion

© 2021. Published by The Company of Biologists under the terms of the Creative Commons Attribution License (https://creativecommons.org/licenses/by/4.0/).

Reviewer 3

Advance summary and potential significance to field

Becker et al. study the effect of modulating Igf1r downstream signalling in eye growth in medaka fish. They show that Igf1r components are expressed in the CMZ in medaka and that pIgf1r positive cells are located in the CMZ and MG cells of the retina. Furthermore, Igf1r inhibitor using an antagonist drug leads to less BrdU+ cells in the CMZ. They then go on and develop a transgenic line that expresses a constitutively active igf1r specifically in the CMZ of medaka, rx2::caigfl1r. The transgenic was validated by showing enhanced pAkt, a downstream effector of Igf1r.
The eyes in rx2::caigfl1r larvae and adult fish are bigger compared to wildtype embryos. Interestingly, the eye organisation in rx2::caigfl1r embryos remains comparable to a wt retina. The authors go on and show data that suggests that the size of the retina in rx2::caigfl1r transgenic is explained by an increase in the number of cells contributed by the CMZ and not from increased proliferation in the central retina not decreased apoptosis. Interestingly the proportion of amacrine and bipolar cells in the inner nuclear layer is significantly increased in rx2::caigfl1r retinas compared to wt conditions. The authors suggest that the cell cycle is shorter in rx2::caigfl1r retinas, with S phase remaining the same and a double of BrdU+ cells observed in the CMZ, which could explain the bigger retinae in rx2::caigfl1r retinas.
To specifically enhance Igf1r activity in the stem cell niche of the CMZ the authors generated a set of cndp transgenic lines, which drive expression in the cells in the CMZ likely to represent the retina stem cells. By using the GaudiRDG system previously developed in their lab, the authors confirm that the cndp driver is expressing the transgenes in the CMZ stem cells as the expected ArCoS are formed and not clonal footprints which would be the consequence of CMZ progenitor cell expression. The authors go on and show that the effect of Igf1r activation over the retina size is exclusively mediated by CMZ progenitor cells and not stem cells as cndp::caigfl1r retinas are not bigger than wt ones.

Comments for the author

The authors have answered most of the suggestions and questions I raised, and the manuscript is significantly improved. However, there are some issues I think could still be resolved.

11. Regarding the sample in the image in Fig2G, the authors provide the retina thickness measurements for this sample compared to the average of these measurements. Besides the retina thickness, could the authors please point out which of the eye diameter/body length, measurements in Table S1 corresponds to this image? This will enable the readers to assess how close or far the measurement of the chosen sample is relative to the median. Can the authors please also include the median and standard deviation for the measurements in Table S1?

12. I am sorry if I was not clear enough when pointing out the use of the concept of homeostasis when referring to optical parameters. In the current version of the manuscript, the authors do specify which are such optical parameters, which I asked for. However, the concept of homeostasis should be applied to physiological parameters such as, for example, blood ion and hormonal composition, or gene expression, which are subject to feedback regulation. I am not sure that the optical parameters referred to by the authors are maintained constant by feedback mechanisms, and so, I am not certain that the concept of homeostasis can be applied to this kind of parameters.

13. The fact that the retina growth can be modified by enhancing or inhibiting Igf signalling hints that the activity of this pathway could mediate the coupling between body and retina growth. However, I still think that the title and statements in this manuscript should be moderated as the data provided does not provide conclusive evidence to state that Igf signalling coordinates retina and body growth. In my view, such coordination would imply some kind of long-term post-embryonic mechanism that enables body and retina growth to be linked.

© 2021. Published by The Company of Biologists under the terms of the Creative Commons Attribution License (https://creativecommons.org/licenses/by/4.0/).

Second revision

Author response to reviewers' comments

The authors have answered most of the suggestions and questions I raised, and the manuscript is significantly improved. However, there are some issues I think could still be resolved.

1. Regarding the sample in the image in Fig2G, the authors provide the retina thickness measurements for this sample compared to the average of these measurements. Besides the retina thickness, could the authors please point out which of the eye diameter/body length, measurements in Table S1 corresponds to this image? This will enable the readers to assess how close or far the measurement of the chosen sample is relative to the median. Can the authors please also include the median and standard deviation for the measurements in Table S1?

> *We thank the reviewer for the comment and have included which fish are depicted in Fig. 2 in the figure legend: "Wt section corresponds to fish 5 and rx2::caigf1r section to fish 16 in Table S1." We have additionally added median and standard deviation to the Tables S1 and S2.*

2. I am sorry if I was not clear enough when pointing out the use of the concept of homeostasis when referring to optical parameters. In the current version of the manuscript, the authors do specify which are such optical parameters, which I asked for. However, the concept of homeostasis should be applied to physiological parameters such as, for example, blood ion and hormonal composition, or gene expression, which are subject to feedback regulation. I am not sure that the optical parameters referred to by the authors are maintained constant by feedback mechanisms, and so, I am not certain that the concept of homeostasis can be applied to this kind of parameters.

> *We thank the reviewer for the clarification of their concern and have accordingly changed "functional homeostasis" to "functional consistency".*

3. The fact that the retina growth can be modified by enhancing or inhibiting Igf signalling hints that the activity of this pathway could mediate the coupling between body and retina growth. However, I still think that the title and statements in this manuscript should be moderated as the data provided does not provide conclusive evidence to state that Igf signalling coordinates retina and body growth. In my view, such coordination would imply some kind of long-term post-embryonic mechanism that enables body and retina growth to be linked.

> *We have changed the title to "Igf signaling couples retina growth with body growth by modulating progenitor cell division".*

Third decision letter

MS ID#: DEVELOP/2020/199133

MS TITLE: Igf signaling couples retina growth with body growth by modulating progenitor cell division

AUTHORS: Clara Becker, Katharina Lust, and Joachim Wittbrodt
ARTICLE TYPE: Research Article

So sorry for the delay in responding about your paper - the revision arrived in the midst of us moving house and so I have a lot of catching up to do! I am, nevertheless happy to tell you that your manuscript has been accepted for publication in Development, pending our standard ethics checks.

© 2021. Published by The Company of Biologists under the terms of the Creative Commons Attribution License (https://creativecommons.org/licenses/by/4.0/).
